# Identification of HIF-2α-regulated genes that play a role in human microvascular endothelial sprouting during prolonged hypoxia in vitro

**DOI:** 10.1007/s10456-016-9527-4

**Published:** 2016-10-03

**Authors:** Tessa D. Nauta, Marloes van den Broek, Sue Gibbs, Tineke C. T. M. van der Pouw-Kraan, Cees B. Oudejans, Victor W. M. van Hinsbergh, Pieter Koolwijk

**Affiliations:** 1 Department of Physiology, Institute for Cardiovascular Research, VU University Medical Center, De Boelelaan 1118, Room 11W53, 1081 HV Amsterdam, The Netherlands; 2 A-Skin Nederland BV, De Boelelaan 1117, 1007 MB Amsterdam, The Netherlands; 3 Department of Dermatology, VU University Medical Center, Amsterdam, The Netherlands; 4 Departments of Oral Cell Biology and Dental Material Sciences, Academic Center for Dentistry Amsterdam (ACTA), University of Amsterdam and Vrije Universiteit, Amsterdam, The Netherlands; 5 Department of Molecular Cell Biology and Immunology, Institute for Cardiovascular Research, VU University Medical Center, Amsterdam, The Netherlands; 6 Department of Clinical Chemistry, VU University Medical Center, Amsterdam, The Netherlands

**Keywords:** Angiogenesis, Hypoxia, HIF-2α, Genome-wide RNA-sequencing

## Abstract

During prolonged hypoxic conditions, endothelial cells change their gene expression to adjust to the low oxygen environment. This process is mainly regulated by the hypoxia-inducible factors, HIF-1α and HIF-2α. Although endothelial cells do not form sprouts during prolonged hypoxic culturing, silencing of HIF-2α partially restores sprout formation. The present study identifies novel HIF-2α-target genes that may regulate endothelial sprouting during prolonged hypoxia. The gene expression profile of primary human microvascular endothelial cells (hMVECs) that were cultured at 20 % oxygen was compared to hMVECs that were cultured at 1 % oxygen for 14 days by using genome-wide RNA-sequencing. The differentially regulated genes in hypoxia were compared to the genes that were differentially regulated upon silencing of HIF-2α in hypoxia. Surprisingly, KEGG pathway analysis showed that metabolic pathways were enriched within genes upregulated in response to hypoxia and enriched within genes downregulated upon HIF-2α silencing. Moreover, 51 HIF-2α-regulated genes were screened for their role in endothelial sprouting in hypoxia, of which four genes ARRDC3, MME, PPARG and RALGPS2 directly influenced endothelial sprouting during prolonged hypoxic culturing. The manipulation of specific downstream targets of HIF-2α provides a new, but to be further evaluated, perspective for restoring reduced neovascularization in several pathological conditions, such as diabetic ulcers or other chronic wounds, for improvement of vascularization of implanted tissue-engineered scaffolds.

**Electronic supplementary material:**

The online version of this article (doi:10.1007/s10456-016-9527-4) contains supplementary material, which is available to authorized users.

## Introduction

Angiogenesis, the formation of new blood vessels through endothelial sprouting, is important for tissue growth, development and proper wound healing. Nevertheless, angiogenesis is associated with several pathological conditions, such as tissue ischemia, solid tumors and adult macular degeneration of the eye [1]. Usually, these disorders are accompanied by loss of adequate blood supply or enhanced metabolic demand, leading to reduced oxygen tension (hypoxia) in the tissue. Not surprisingly, hypoxia is considered to be one of the most potent initiators of angiogenesis in vitro and in vivo [2–4] through stabilization of the transcription factor hypoxia-inducible factor-1α (HIF-1α) and subsequent induction of vascular endothelial growth factor (VEGF) [5, 6]. Nonetheless, in chronic hypoxic tissues often a resistance to induction of neovascularization is observed [3, 7–11] and limited expression of HIF-1 has been observed in chronic hypoxic human leg tissue [12]. Therefore, there is a need of stimulating neovascularization and temporarily overcoming the endogenous inhibitory factors that prevent induction of angiogenesis in severely hypoxic tissues.

Despite the progress in understanding the mechanisms and factors regulating angiogenesis [13, 14], the effects of long-term hypoxia on angiogenesis regulation and in particular the behavior of endothelial cells are still poorly understood. Hypoxia rapidly induces large amounts of VEGF-A via HIF-1 activation in all tissue cells, thus supplying a major angiogenesis-inducing factor in the environment. It activates VEGF receptors on the endothelium which induce proliferation and—often stimulated by additional inflammatory factors—cell migration and invasion. However, the transcriptional response of endothelial cells is more complicated and involves—together with additional factors—the involvement of HIF-1α and HIF-2α, which display a partly different spectrum of activation. HIF-1α has been studied extensively and induces mainly tortuous and leaky newly formed vascular structures that are not adequately perfused [15, 16], while HIF-2α (EPAS1), which is abundantly expressed in endothelial cells, induces stabilization of endothelial microvessels, and—in lung microvessels—improves endothelial barrier function [17]. Indeed, endothelial-specific deletion of HIF-2α pointed to a role of HIF-2α in the regulation of angiogenesis in mice [18, 19], which was suggested to occur through the induction of the Dll4/Notch signaling pathway. These data suggest that the endothelial cell has its own program to respond adequately to hypoxia, i.e., in such a way that finally a mature, sealed and stabilized new microvascular network is formed. Excess of HIF-1α response, as observed in many tumors, leads to abundant tortuous but not or suboptimal functioning microvessels. As the activation of HIF-1α and HIF-2α is in part transient and HIF-2α tends to be longer expressed, the balance between sprouting and stabilization may shift during prolonged hypoxia. This may underlie our previous observation that prolonged hypoxia reduced the ability of endothelial cells to form sprouts. This was partly related to a reduction of urokinase–plasminogen activator generation, which reduces pericellular proteolysis by the uPA/uPAR/plasmin pathway and—independently—could be partially relieved by HIF-2α si-RNA [20]. However, the si-HIF-2α-dependent restoration of sprouting in this study occurred independent of Dll4/Notch signaling.

The current study investigates which genes and pathways are differentially regulated in prolonged hypoxia and downstream of HIF-2α in conditions that were previously shown to be favorable for induction of endothelial tubules, i.e., hMVECs seeded on a 3D-fibrin matrix and stimulated by VEGF-A/TNFα [20, 21]. Many hypoxia-responsive genes have been identified using microarrays in different tumor cell lines [22–28] and human endothelial cells [4, 29]. However, only short-term hypoxia (16–72 h) under basal conditions was evaluated, but not prolonged hypoxic conditions (14 days). Therefore, we studied gene expression after long-term hypoxia and with and without silencing of HIF-2α. By using genome-wide RNA-sequencing, 51 genes were identified in VEGF-A/TNFα-stimulated cells that were regulated in a reversed way in hypoxia or by HIF-2α silencing and were screened for their ability to regulate sprouting of human endothelial cells in a 3D fibrin matrix.

## Materials and methods

### Cell culture

The study was executed in accordance with the Declaration of Helsinki and was approved by the University Human Subjects Committee of the VU University Medical Center. Written informed consent was obtained from all donors in accordance with the institutional guidelines. Human microvascular endothelial cells (hMVECs) were isolated from foreskin, kindly provided by the Department of Dermatology (VUmc, Amsterdam), cultured and characterized (CD31, vWF, Ulex europaeus lectin-1 binding, VE-cadherin) as previously described [30, 31]. hMVECs were cultured on 1 % gelatin-coated culture plates in culture medium consisting of Medium 199 supplemented with 100 U/mL penicillin and 100 mg/mL streptomycin (p/s), 2 mM l-glutamine (all Lonza, Verviers, Belgium), 5 U/mL heparin (Leo Pharmaceutical Products, Weesp, The Netherlands), endothelial cell growth factor (ECGF, crude extract from bovine brain), 10 % heat-inactivated human serum (HSi, Life Technologies) and 10 % heat-inactivated newborn calf serum (NBCSi, Lonza). Medium was changed every 48 h. Confluent cells were washed with 0.5 mM EDTA (Merck Millipore) in HBSS, trypsinized (0.05 % trypsin in EDTA/HBSS, both Lonza) and seeded in a 1:3 density. Cells were cultured at 37 °C in a water-saturated atmosphere of 95 % air and 5 % CO_2_. hMVECs were used until passage 10.

### Hypoxic cell culture

Hypoxic cell culture conditions were maintained inside a custom designed hypoxic workstation (T.C.P.S., Rotselaar, Belgium), with a CO_2_ and O_2_ controlled (via injection of N_2_), humidified incubator (Sanyo, Etten-leur, The Netherlands), placed inside a T4 glovebox (Jacomex, Dagneux, France) equipped with an O2X1 oxygen transmitter (GE Panametrics, Billerica, USA). The oxygen concentration inside the incubator was continuously monitored with an internal zirconia sensor and periodically checked with O_2_ test tubes (Drager Safety, Zoetermeer, The Netherlands). To prevent re-oxygenation during hypoxic culture, all media and buffers were preincubated for 4 h before use. For the long-term hypoxic culture of hMVECs, isolates were cultured for 2 passages (~14 days) inside the hypoxic workstation.

### In vitro tube formation assay

3D human tube formation was evaluated as previously described [21]. 2 mg/mL fibrinogen (Stago bnl, Leiden, The Netherlands) was dissolved in M199 medium + p/s. Thrombin (0.05 U/mL) was added to the fibrinogen solution and 100 μL was immediately added to wells of a 96-well plate. For polymerization, plates were incubated for 1 h at room temperature followed by 1 h at 37 °C. Thrombin was inactivated by addition of serum-supplemented culture (SSC) medium consisting of Medium 199 with p/s supplemented with 10 % HSi, 10 % NBCSi and 2 mM l-glutamine. hMVECs, precultured for 14 days at 20 % or 1 % oxygen, were seeded in a confluent density on top of the fibrin matrices. After 24 h, and subsequently at 48-h intervals, the hMVECs were stimulated with SSC medium with 10 ng/mL tumor necrosis factor-α (TNFα, Sigma, St Louis, USA) and 25 ng/mL vascular endothelial growth factor (VEGF, Invitrogen, Carlsbad, USA) or 10 ng/mL TNFα and 10 ng/mL fibroblast growth factor-2 (FGF-2, Preprotech, London, UK). The experiments were terminated by fixation with 2 % paraformaldehyde/HBSS for 2 h at room temperature. The formation of tube-like structures from hMVECs into the fibrin matrices was analyzed by phase contrast microscopy and Optimas image analysis software.

### Transfection with si-RNA

3 × 10^5^ hMVECs were transfected with 25 nM of indicated si-RNA (si-HIF-2α was custom designed and obtained from Qiagen (Venlo, The Netherlands)) and 51 genes for screening were ordered as Cherry-pick Library from GE Dharmacon (Lafayette, CO) using DharmaFECT transfection reagent Type 1. In short, hMVECs were transfected with 2 mL 10 % HSi/M199 containing 2.5 μL DharmaFECT transfection reagent Type 1 and si-RNA. 18 h after transfection, cells were refreshed with culture medium to start the experiment. In prolonged hypoxia experiments, the procedure was performed continuously in a 1 % oxygen atmosphere.

### RNA isolation and genome-wide RNA-sequencing

hMVECs were cultured for 14 days at normoxic or hypoxic conditions. Upon confluency, cells were starved for 18 h in SSC medium and afterward stimulated with VEGF (10 ng/mL) and TNFα (10 ng/mL) in SSC medium for 24 h. Total RNA was isolated by using the RNeasy Mini kit according to manufacturer’s protocol without the DNAse treatment (Qiagen).

4 μg of RNA/sample with an RIN ≥ 9.8 was subjected to a double round of poly-A mRNA purification, fragmented, and primed for cDNA library synthesis using the TruSeq RNA sample preparation kit (FC-122-1001). All procedures were done according to the manufacturer’s instructions (Illumina). Following validation (Agilent 2100 Bioanalyzer, DNA High Sensitivity) and normalization (AUC 200- to 500-bp fragments), samples were clustered (TruSeq paired-end cluster kit v3-cBot-HS, PE-401-3001) followed by paired-end sequencing (100 bp; TruSeq SBS kit v3-HS 200 cycles, FC-401-3001) on a HiSeq2500. RNA-Seq reads were aligned to the preassembled reference genome (Illumina iGenome, data source UCSC assembly hg19; February 2009) using TopHat (version 2.0.9) in combination with Bowtie (version 2.1.0) and SAMtools (version 0.1.18) using the default settings [32]. Transcript assembly, abundance estimation (defined as fragments per kilobase of exon per million fragments mapped; FPKM) and differential expression were performed by sequential analysis of TopHat output (accepted_hits.bam). For this, transcripts were assembled using Cufflinks (version 2.1.1) under conditions (RABT assembly) [33] permitting the identification of novel unannotated transcripts (transcripts.gtf) and with correction for fragment bias to account for biases in library preparation [34]. The assemblies to be compared were merged (Cuffmerge), generating a transcript index (merged.gtf). Subsequently, differential analysis of significant changes in transcript expression, splicing and promoter use was performed (Cuffdiff) in the different transfection couples (e.g., hypoxia vs. normoxia).

### Quantitative real-time PCR

Quantitative real-time polymerase chain reaction (qRT-PCR) was performed using identical RNA as used for the genome-wide RNA-sequencing. Copy DNA (cDNA) was synthesized of 1 μg RNA using the Cloned AMV First Strand cDNA Synthesis Kit from Invitrogen with poly(T)primers. β-2-microglobulin was used as the endogenous reference gene. To measure gene expression, qRT-PCR was performed in duplicate wells using SYBR Green in an ABI 7500 sequence detection system (Applied Biosystems, Foster City, USA). Briefly, 10 μL mix was prepared using 20 ng cDNA, 100 nM forward primer, 100 nM reverse primer and MESA Green QPCR Mastermix Plus for Sybr Assay (Eurogentec, Seraing, Belgium). Protocol: 2 min 50 °C, 10 min 95 °C and 40 cycles (0:15 min 95 °C, 1:00 min 60 °C) and dissociation curve. Relative expression levels of target genes (see also Supplementary Table 1) were calculated with the reference gene β-2-microglobulin with the comparative *C*_q_ method, as described by Wong et al. [35].

### Tube formation screening

Four independent hMVEC donors were cultured in normoxia until confluency, pooled together and seeded on top of 3D fibrin matrices. The hMVECs were transfected with specific si-RNA against the individual genes 4 h after seeding and 18 h later stimulated with VEGF-A/TNFα and transferred to hypoxia. Seven days after stimulation with VEGF-A/TNFα, two researchers evaluated the number of sprouts independently by eye and only the genes that were scored as more or less sprouts compared with the scrambled control by both researchers were selected for further investigation. In addition, invading cells and the formation of tubular structures of endothelial cells in the 3D fibrin matrix were analyzed by phase contrast microscopy. The total length of tube-like structures of triplicate wells was measured using 4 randomly chosen semi-dark field pictures/well using a Nikon FXA microscope equipped with a monochrome CCD camera (MX5). After threshold setting and skeletonization, the sprout formation was expressed as mm/cm^2^ using Optimas image analysis software (Adept Turnkey, Sydney, Australia).

### Statistical analysis

Statistical analysis was performed using one-way ANOVA or TWO-way ANOVA with Bonferroni post hoc test. Numbers of replicates and significant *P* values are indicated in the text or figures. *P* < 0.05 was considered significant. Results are shown as mean ± SEM or ±range.

Statistical analysis on genome-wide RNA-sequencing data was performed using significance analysis of microarrays (SAM) [36]. Genes that were expressed at significantly different levels were defined by a FDR of <5 % and fold change >1.5. For visualization of protein–protein interactions and pathway analysis (Kyoto Encyclopedia of Genes and Genomes (KEGG) [37], STRING10 analysis was used [38].

## Results

### HIF-2α regulates endothelial sprouting in prolonged hypoxia

**Fig. 1:**
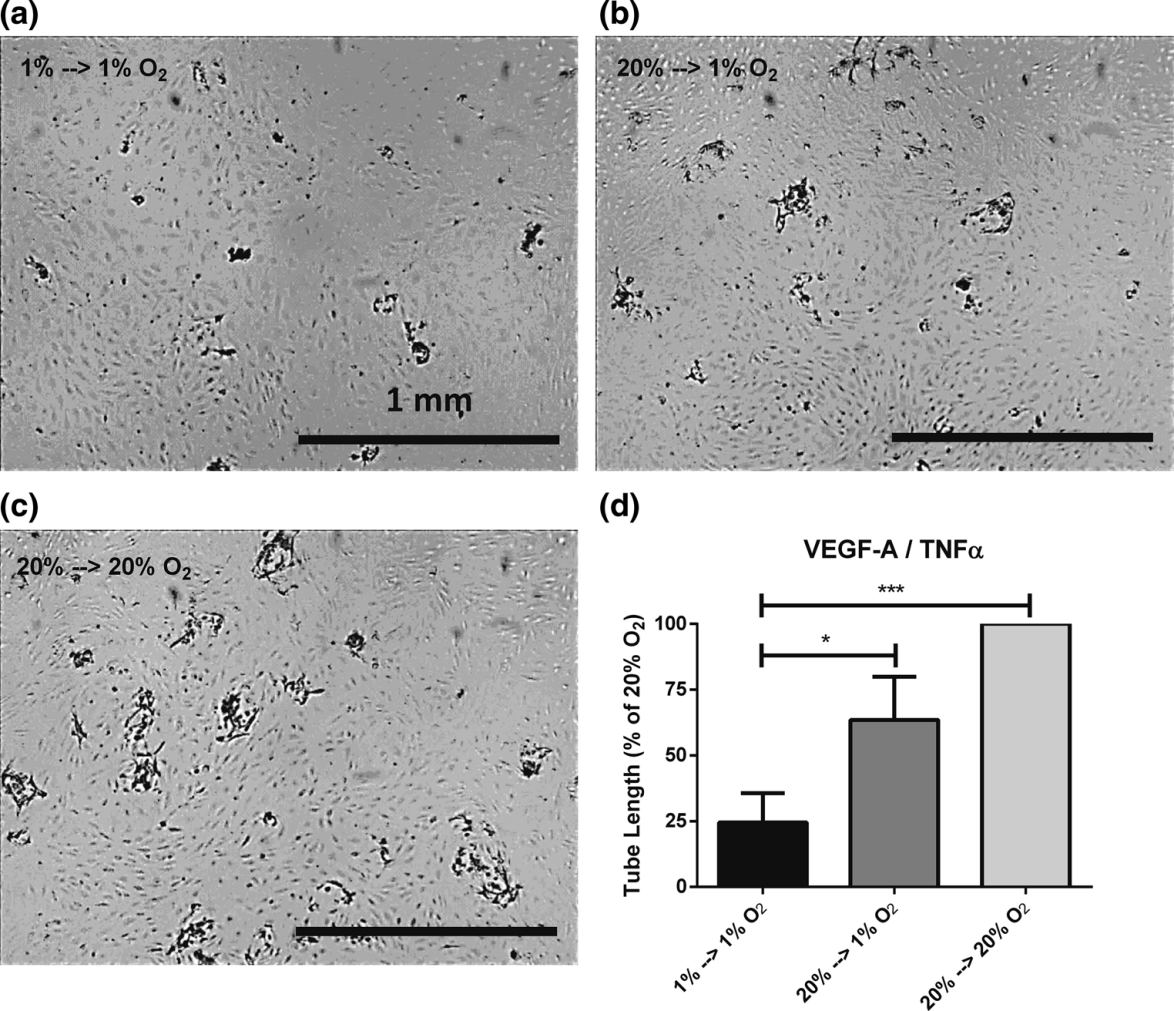
Prolonged hypoxia inhibits endothelial sprouting into 3D fibrin matrices. hMVECs were precultured at 1 % or 20 % O_2_ for 14 days before seeded on top of 3D fibrin matrices. Subsequently, the hMVECs were stimulated with the combination of VEGF-A/TNFα (*n* = 5 donors in 7 experiments) either at 20 % or 1 % O_2_ (each in triplo). **a**–**c** Representative photographs are shown of hMVECs 7 days after seeding and stimulation with VEGF-A/TNFα. The *scale bars* represent 1 mm. Photographs are focused on the sprouts. **d** Tube length was quantified by using Optimas software and expressed as percentage of 20 % O_2_ with SEM. For statistical analysis, one-way ANOVA with Bonferroni post hoc test was used (*p < 0.05; ***p < 0.001)

Endothelial cells formed sprouts into a 3D fibrin matrix under normoxic conditions within 7 days upon stimulation with VEGF-A/TNFα. Sprout formation was decreased when the assay was performed under hypoxic conditions for 7 days (20 % O_2_ → 1 % O_2_) (Fig. 1), similarly as we previously showed [20]. Furthermore, when hMVECs were precultured for 14 days in hypoxic conditions (1 % O_2_/94 % N_2_/5 % CO_2_) without reoxygenation, sprout formation was severely hampered (Fig. 1), independent of cell proliferation [20]. Silencing HIF-2α with si-RNA partially restored endothelial sprouting in hypoxia and increased sprout formation in normoxia, indicating an inhibitory role of HIF-2α during sprout formation by prolonged hypoxic and normoxic cultured hMVECs (showed below).

### Identifying oxygen-regulated genes and pathways

In order to identify the underlying HIF-2α mechanisms and target genes that may be involved in the inhibition of the VEGF-A/TNFα-induced endothelial sprouting during prolonged hypoxia, the endothelial transcriptome was explored. Similar culture conditions were used as the in vitro 3D tube formation assay, e.g., 14 days of normoxic or hypoxic preculturing followed by stimulation with VEGF-A/TNFα.

Gene expression profiling of hMVEC cultures from four independent donors was performed by genome-wide RNA-sequencing analysis, and the Tuxedo pipeline with default settings [32] was used. Data were analyzed as paired using SAM. The individual donors expressed between 12,000 and 13,000 known genes. In total, 2335 genes were significantly (FDR < 5 %) differentially regulated after 24-h stimulation with VEGF-A/TNFα in hypoxia-precultured hMVECs compared with normoxia-precultured hMVECs. Of these 2335 genes, 501 upregulated and 333 downregulated genes had an absolute fold change of >1.5. The top 25 most upregulated and top 25 most downregulated genes in prolonged hypoxia, ranked on basis of fold change, are shown in Table 1. These genes include PHD3 (EGLN3), HIF-3α (HIF3A) and GLUT1 (SLC2A1), which are known to be upregulated in hypoxia in different cell types including endothelial cells [4, 22–24, 29, 39]. Even though VEGF-A was not present within the top 25 genes, it was significantly upregulated in hypoxia (3.5-fold). The list of all oxygen-regulated genes can be found in Supplementary Table 2.

**Table 1:**
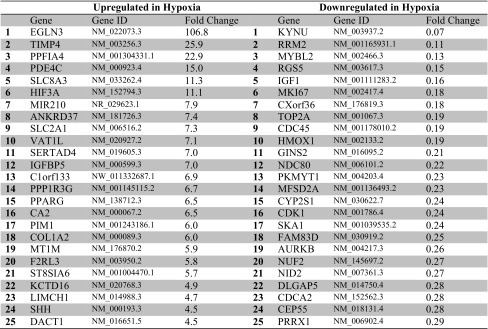
Top 25 genes with significantly induced or repressed gene expression in hypoxia-precultured hMVECs after VEGF-A/TNFα stimulation

The relative gene expression was compared with the gene expression of hMVECs precultured in normoxia and stimulated with VEGF-A/TNFα (*n* = 4 independent donors)

**Fig. 2:**
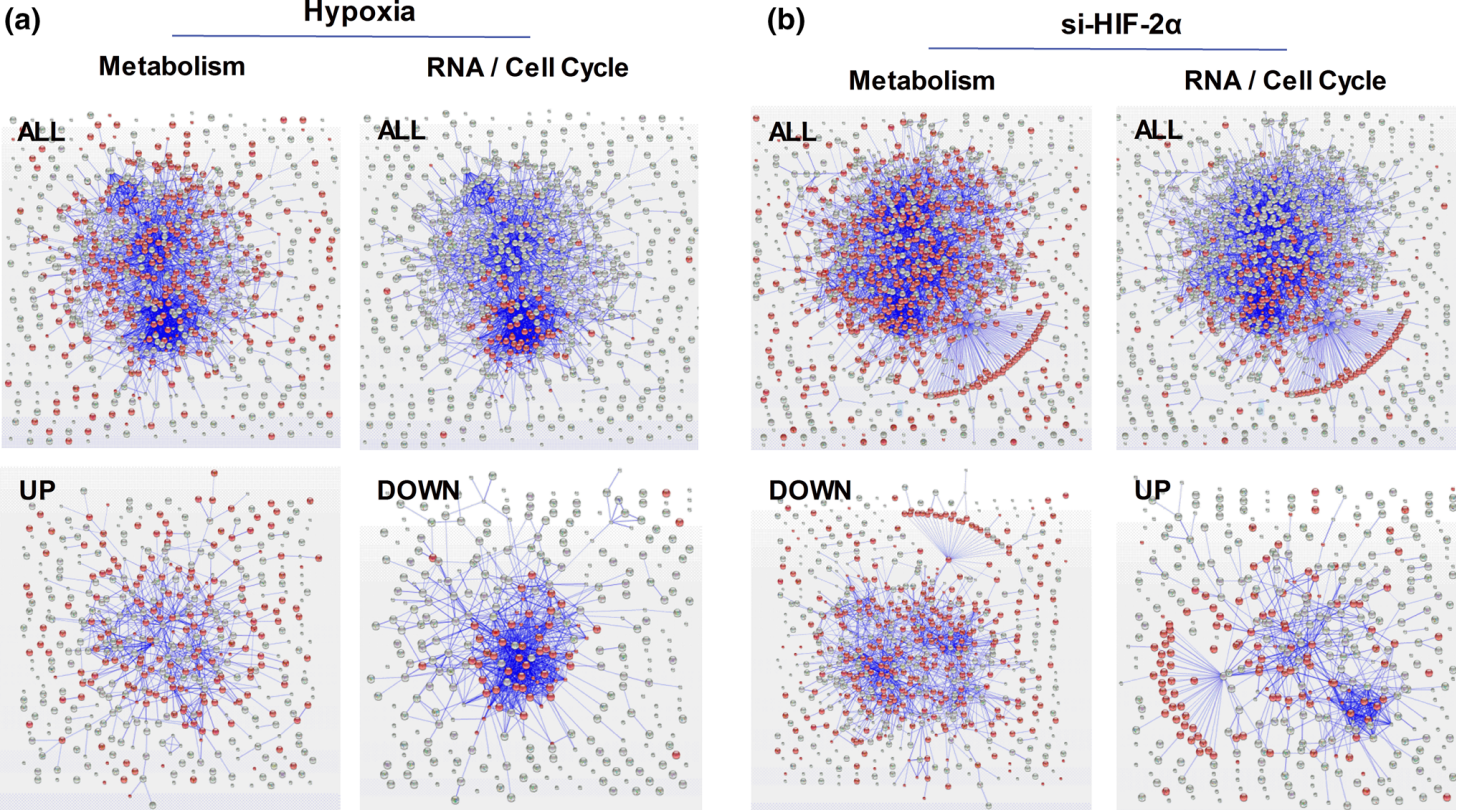
Significantly regulated genes by hypoxia or si-HIF-2α are involved in metabolism or cell cycle. Genes that were differentially regulated (FDR < 5 %, absolute fold difference >1.5) in hypoxia (**a**) or upon HIF-2α silencing (**b**) were clustered based on protein–protein interactions. The nodes represent the proteins and a shared function of the proteins are shown as interconnecting blue lines. The thickness of these *lines* indicates the confidence of the association. All genes (*upper panels*), or only the upregulated or the downregulated genes (*lower panels*) were clustered based on GO biological processes involved in metabolic or RNA/cell cycle pathways; genes involved in these pathways are indicated in *red*. (Color figure online)

To investigate which pathways are important in hypoxia signaling, we clustered the oxygen-regulated genes on the basis of protein–protein interactions (Fig. 2a) and subsequently categorized the genes into KEGG pathways important in angiogenesis (Table 2). The angiogenic pathways that were significantly altered by prolonged hypoxia in the presence of VEGF-A/TNFα included the HIF-1 signaling pathway, and several metabolic, cell cycle and amino acid biosynthesis pathways. When the upregulated and the downregulated genes by hypoxia were clustered separately and categorized into KEGG pathways, the upregulated genes showed an enrichment within cytokine–cytokine receptor interactions, such as TNFα, TGF-β and MAPK signaling pathways, and several metabolic pathways, such as glycolysis/gluconeogenesis, and carbon-, fructose- and mannose metabolism (Table 2; Fig. 2a). In contrast, the downregulated genes were categorized mainly in cell cycle, DNA replication and the p53 signaling pathways (Table 2; Fig. 2a). Taken together, these data show that 834 genes are significantly differentially regulated in hypoxia. Many of the upregulated genes are enriched in metabolic pathways, whereas many of the downregulated genes are enriched in cell cycle pathways.

**Table 2:** Hypoxia pathway analysis

All genes (834)	Upregulated genes (501)	Downregulated genes (333)
HIF-1 signaling pathway^(5.7E−07)^	Cytokine–cytokine receptor interaction^(3.8E−06)^	Cell cycle^(5.8E−07)^
p53 signaling pathway^(9.3E−06)^	Glycolysis/gluconeogenesis^(7.7E−05)^	p53 signaling pathway^(1.0E−05)^
Cytokine–cytokine receptor interaction^(1.1E−04)^	HIF-1 signaling pathway^(1.6E−04)^	HIF-1 signaling pathway^(1.4E−03)^
Cell cycle^(3.1E−04)^	Biosynthesis of amino acids^(2.3E−04)^	Glycine, serine and threonine metabolism^(3.4E−03)^
Biosynthesis of amino acids^(4.1E−04)^	TNF signaling pathway^(8.9E−02)^	Homologous recombination^(9.0E−03)^
Glycolysis/gluconeogenesis^(5.5E−04)^	Inflammatory mediator regulation of TRP channels^(1.7E−03)^	Arginine and proline metabolism^(1.3E−02)^
Inflammatory mediator regulation of TRP channels^(1.2E−03)^	Carbon metabolism^(2.8E−03)^	VEGF signaling pathway^(1.5E−02)^
Cysteine and methionine metabolism^(2.7E−03)^	Calcium signaling pathway^(2.8E−03)^	DNA replication^(1.8E−02)^
FoxO signaling pathway^(2.9E−03)^	Pentose phosphate pathway^(3.0E−03)^	Biosynthesis of unsaturated fatty acids^(4.4E−02)^
TNF signaling pathway^(3.2E−03)^	Hedgehog signaling pathway^(6.3E−03)^	
Glycine, serine and threonine metabolism^(4.0E−03)^	Fructose and mannose metabolism^(6.4E−03)^	
Proteoglycans in cancer^(6.1E−03)^	FoxO signaling pathway^(7.5E−03)^	
Carbon metabolism^(7.1E−03)^	Cysteine and methionine metabolism^(9.8E−03)^	
Pentose phosphate pathway^(1.8E−02)^	Neuroactive ligand–receptor interaction^(1.2E−02)^	
Calcium signaling pathway^(2.1E−02)^	Galactose metabolism^(3.3E−02)^	
Arginine and proline metabolism^(2.5E−02)^	Proteoglycans in cancer^(3.4E−02)^	
VEGF signaling pathway^(2.9E−02)^	TGF-beta signaling pathway^(3.5E−02)^	
Rap1 signaling pathway^(3.3E−02)^	MAPK signaling pathway^(3.6E−02)^	
Fructose and mannose metabolism^(3.6E−02)^	NOD-like receptor signaling pathway^(4.3E−02)^	
Cell adhesion molecules (CAMs)^(4.4E−02)^	Glycosaminoglycan biosynthesis—keratan sulfate^(4.7E−02)^	
Hedgehog signaling pathway^(4.7E−02)^	Apoptosis^(4.8E−02)^	
Protein digestion and absorption^(3.8E−02)^		

The KEGG pathways involved in angiogenesis or metabolism with a *p* value of <0.05 are shown. Significance per pathway is shown in parenthesis

### Identifying HIF-2α-regulated genes and pathways

HIF-2α silencing partially restored the endothelial sprouting (see below, Fig. 5a) in long-term hypoxia. HIF-2α mRNA was significantly silenced with si-RNA, while HIF-1α mRNA was not affected (Supplementary Table 3). HIF-3α mRNA, in contrast, was significantly downregulated with an si-RNA of HIF-2α, but this is probably a nonspecific effect as scrambled si-RNA similarly decreased HIF-3α mRNA expression (Supplementary Table 3). The RNA-seq data revealed in total 6757 significantly (FDR < 5 %) differentially regulated genes upon HIF-2α silencing compared with prolonged hypoxia (untransfected). However, 33 % of these genes were also significantly regulated by transfection with scrambled si-RNA and were excluded for further analysis. Of the remaining genes, 449 upregulated and 715 downregulated genes had an absolute fold change of >1.5. The 25 most upregulated and most downregulated genes upon HIF-2α silencing, including HIF-2α mRNA (EPAS1), ranked on basis of fold change, are shown in Table 3. The list of all HIF-2α-regulated genes can be found in Supplementary Table 4.

**Table 3:**
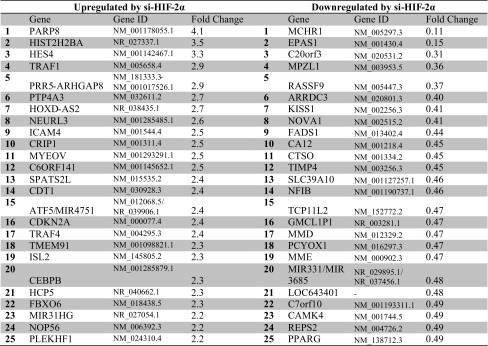
Top 25 genes with significantly induced or repressed gene expression upon HIF-2α knock-down, but not upon scrambled si-RNA transfection, in hypoxia-precultured hMVECs after VEGF-A/TNFα stimulation

The relative gene expression was compared with the gene expression of hMVEC precultured in hypoxia and stimulated with VEGF-A/TNFα (*n* = 4 independent donors)

Clustering of all si-HIF-2α-regulated genes based on protein–protein interactions is shown in Fig. 2b, and these genes were subsequently categorized into KEGG pathways important in angiogenesis (Table 4). In contrast to the many downregulated genes by hypoxia that were involved in RNA/cell cycle (Fig. 2a), the genes involved in cell cycle, ribosome biogenesis and RNA transport were upregulated upon HIF-2α silencing (Table 4; Fig. 2b). Moreover, metabolic genes were upregulated in hypoxia (Fig. 2a), but downregulated upon HIF-2α silencing (Table 4; Fig. 2b). These findings indicate that 1164 genes are significantly differentially regulated upon silencing of HIF-2α with si-RNA. Opposite to the oxygen-regulated genes, many of the si-HIF-2α upregulated genes are enriched in cell cycle/RNA processes, while the si-HIF-2α downregulated genes are involved in multiple metabolic pathways.

**Table 4:** HIF-2α pathway analysis

All genes (1164)	Upregulated genes (449)	Downregulated genes (715)
Carbon metabolism^(5.7E−04)^	Ribosome biogenesis in eukaryotes^(4.1E−03)^	Fatty acid metabolism^(4.0E−05)^
Biosynthesis of unsaturated fatty acids^(8.2E−04^)	Vitamin B6 metabolism (^6.1E−03)^	Biosynthesis of unsaturated fatty acids^(6.6E−05)^
Fatty acid metabolism^(1.3E−03)^	MAPK signaling pathway^(6.6E−03)^	Metabolic pathways^(9.4E−05)^
Citrate cycle (TCA cycle)^(4.1E−03)^	RNA transport^(3.9E−02)^	Carbon metabolism^(2.6E−04)^
MAPK signaling pathway^(4.1E−03)^	Cell cycle^(4.1E−02)^	PPAR signaling pathway^(2.7E−03)^
Metabolic pathways^(7.5E−03)^	Estrogen signaling pathway^(4.5E−02)^	Citrate cycle (TCA cycle)^(2.7E−03)^
GnRH signaling pathway^(9.5E−03)^		Regulation of actin cytoskeleton^(7.3E−03)^
p53 signaling pathway^(1.1E−02)^		p53 signaling pathway^(8.6E−03)^
Oxytocin signaling pathway^(1.2E−02)^		Axon guidance^(1.2E−03)^
Sphingolipid metabolism^(1.5E−02)^		Osteoclast differentiation^(1.2E−02)^
cGMP-PKG signaling pathway^(1.6E−02)^		Focal adhesion^(1.3E−02)^
Axon guidance^(2.1E−02)^		Ether lipid metabolism^(1.3E−02)^
Vascular smooth muscle contraction^(2.8E−02)^		Ribosome^(1.6E−02)^
Glycerophospholipid metabolism^(3.2E−02)^		Fatty acid degradation^(1.7E−02)^
Propanoate metabolism^(3.2E−02)^		Valine, leucine and isoleucine degradation^(1.9E−02)^
Valine, leucine and isoleucine degradation^(3.6E−02)^		Sphingolipid metabolism^(2.4E−02)^
PPAR signaling pathway^(3.7E−02)^		Propanoate metabolism^(2.5E−02)^
Vitamin B6 metabolism^(4.1E−02)^		Starch and sucrose metabolism^(3.1E−02)^
Wnt signaling pathway^(4.1E−02)^		GnRH signaling pathway^(3.4E−02)^
Osteoclast differentiation^(4.4E−02)^		Endocytosis^(4.1E−02)^
VEGF signaling pathway^(4.7E−02)^		Oxytocin signaling pathway^(4.4E−02)^
Pantothenate and CoA biosynthesis^(4.9E−02)^		Sulfur metabolism^(4.7E−02)^
		Ras signaling pathway^(4.8E−02)^
		Ubiquitin-mediated proteolysis^(4.8E−02)^

The KEGG pathways involved in angiogenesis or metabolism with a *p* value of <0.05 are shown. Significance per pathway is shown in parenthesis

### Identification of genes that may regulate endothelial sprouting in prolonged hypoxia

To identify genes that may be involved in the regulation of endothelial sprouting during prolonged hypoxia, we set multiple criteria in our RNA-seq data: (1) genes should be significantly regulated (FDR < 5 % and fold change >1.5) in hypoxia, (2) genes should be significantly regulated upon HIF-2α silencing but not by scrambled si-RNA, and (3) genes should be regulated in opposite directions in hypoxia and after si-HIF-2α. These criteria resulted in 51 genes (Table 5), as potential candidates that may regulate endothelial sprouting into fibrin in hypoxia. Clustering of the genes based on protein–protein interactions did not reveal a significant enriched pathway.

**Table 5:**
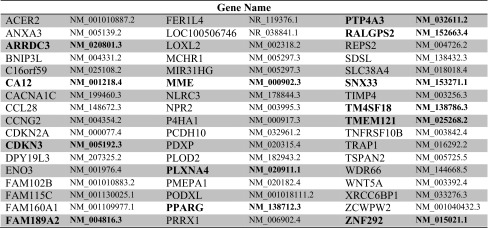
51 genes were selected from genome-wide RNA-sequencing

Fifty-one genes were significantly differentially regulated in hypoxia and upon HIF-2α silencing in opposite directions, but not by transfection with scrambled si-RNA. Through an initial sprouting screening, 13 genes were selected that altered endothelial sprouting upon silencing; these genes are highlighted in bolt. The genes that were upregulated in prolonged hypoxia are indicated in red, and the genes that are downregulated in prolonged hypoxia are indicated in green

To examine which of these genes played a role in endothelial sprouting in hypoxia, an initial screening using specific si-RNAs of these genes was performed with a pool of four independent hMVEC donors. From this initial screening, 13 genes were selected as positive candidates, including 9 genes that were upregulated in hypoxia (highlighted in red in Table 5) and therefore potential inhibitors of sprouting, and 4 genes that were downregulated in hypoxia and therefore possible stimulators of angiogenesis (highlighted in green in Table 5). Taken together, from the RNA-seq51 genes are significantly differentially regulated in hypoxia and upon HIF-2α silencing in opposite directions, but not by scrambled si-RNA. Moreover, the initial screening using specific si-RNAs reveals 13 genes that are selected as potential regulators of angiogenesis.

### Involvement of the 13 genes in regulating endothelial sprouting

**Fig. 3:**
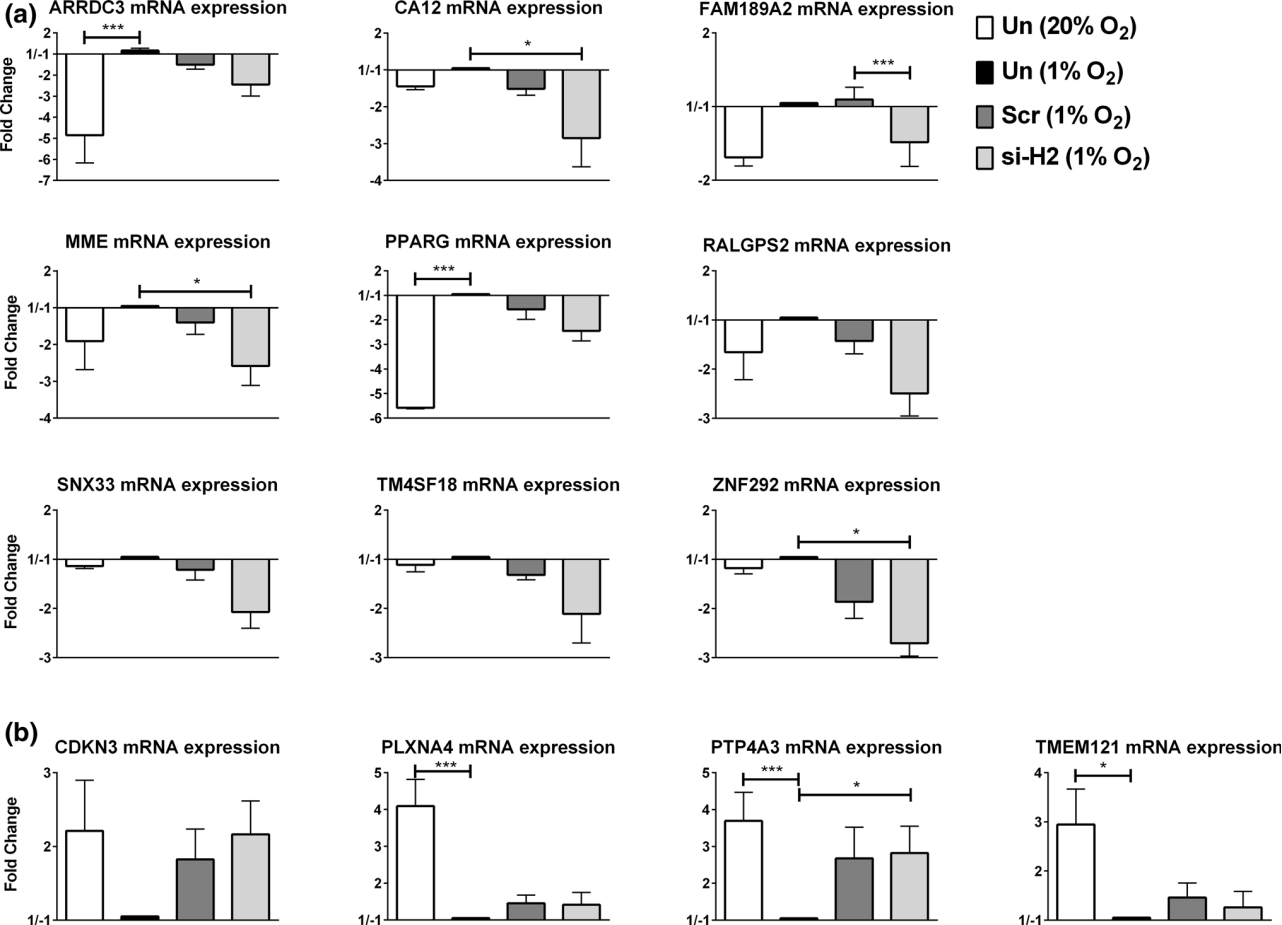
Relative mRNA expression of 13 genes that were selected after the screening. hMVECs were cultured in normoxia for 14 days, not transfected and stimulated for 24 h with VEGF-A/TNFα in normoxia [Un (20 % O_2_), *white bar*] or cultured in prolonged hypoxia for 14 days, not transfected and stimulated for 24 h with VEGF-A/TNFα in hypoxia [Un (1 % O_2_), *black bar*]. Moreover, hMVECs were cultured in prolonged hypoxia for 14 days, transfected with si-HIF-2α [si-H2 (1 % O_2_), *light gray bar*] or scrambled [scr (1 % O_2_), *dark gray bar*] and stimulated for 24 h with VEGF-A/TNFα in hypoxia. mRNA was isolated for analysis by qRT-PCR, and the relative mRNA levels of the 13 candidate genes were expressed as mean fold change with SEM (*n* = 4 independent donors). Data were normalized to 1 % O_2_. **a** Nine genes that were upregulated in the RNA-seq analyses. **b** Four genes that were downregulated in the RNA-seq analyses. For statistical analysis, two-way ANOVA with Bonferroni post hoc test was used (*p < 0.05; ***p < 0.001)

**Fig. 4:**
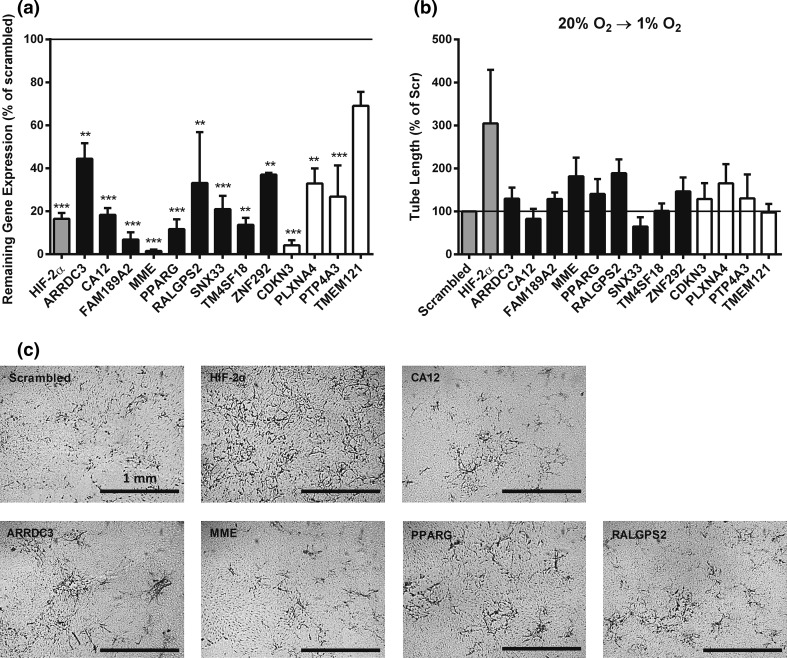
Transfection efficiency of candidate genes and effect of their silencing on endothelial sprouting in short-term hypoxia. hMVECs were precultured at 20 % oxygen and silenced with one of the 13 genes selected from the screening. **a** mRNA was isolated to analyze the knock-down efficiency of si-RNA. The knock-down efficiency was expressed as mean with range (*n* = 2 independent donors), and scrambled transfection was set as 100 % (horizontal line). **b** hMVECs were seeded on top of fibrin matrices before transfection with si-RNA. Subsequently, the hMVECs were stimulated with the combination of VEGF-A and TNFα and transferred to hypoxia. Tube length of hMVECs 7 days after stimulation with VEGF-A/TNFα was quantified by using Optimas software and expressed as percentage of Scrambled with SEM (*n* = 3 independent donors, each in triplicate). **c** Representative photographs are shown of hMVECs 7 days after seeding and stimulation with VEGF-A/TNFα. The scale bars represent 1 mm. Photographs are focused on the sprouts. The genes that were upregulated in prolonged hypoxia are expressed as black bars, and the genes that were downregulated in prolonged hypoxia are expressed as white bars. For statistical analysis, one-way ANOVA with Bonferroni post hoc test was used (**p < 0.01; ***p < 0.001)

The changes in relative mRNA expression of the selected genes were confirmed with quantitative real-time PCR (Fig. 3a, b). The pattern of hypoxia down-/si-HIF-2α up-regulated genes was consistently observed (Fig. 3a), but the hypoxia up-/si-HIF-2α down-regulated genes were only found in 2 out 4 genes and no differences were seen between si-HIF-2α and Scr (Fig. 3b). Next, the role of the 13 selected genes from the initial screening in sprout formation was investigated in independent hMVEC donors instead of a pooled hMVEC batch, in short-term hypoxia, prolonged hypoxia and normoxia. Through efficient knock down of the mRNA with specific si-RNAs (Fig. 4a), we found that silencing of ARRDC3, CDKN3, FAM189A2, MME, PLXNA4, PPARG, PTP4A3, RALGPS2 and ZNF292 resulted in an increased sprouting of more than 25 % compared with scrambled si-RNA. Silencing of SNX33 resulted in a decreased sprouting of more than 25 % compared with scrambled in short-term (7 days) hypoxia (Fig. 4b, c).

**Fig. 5:**
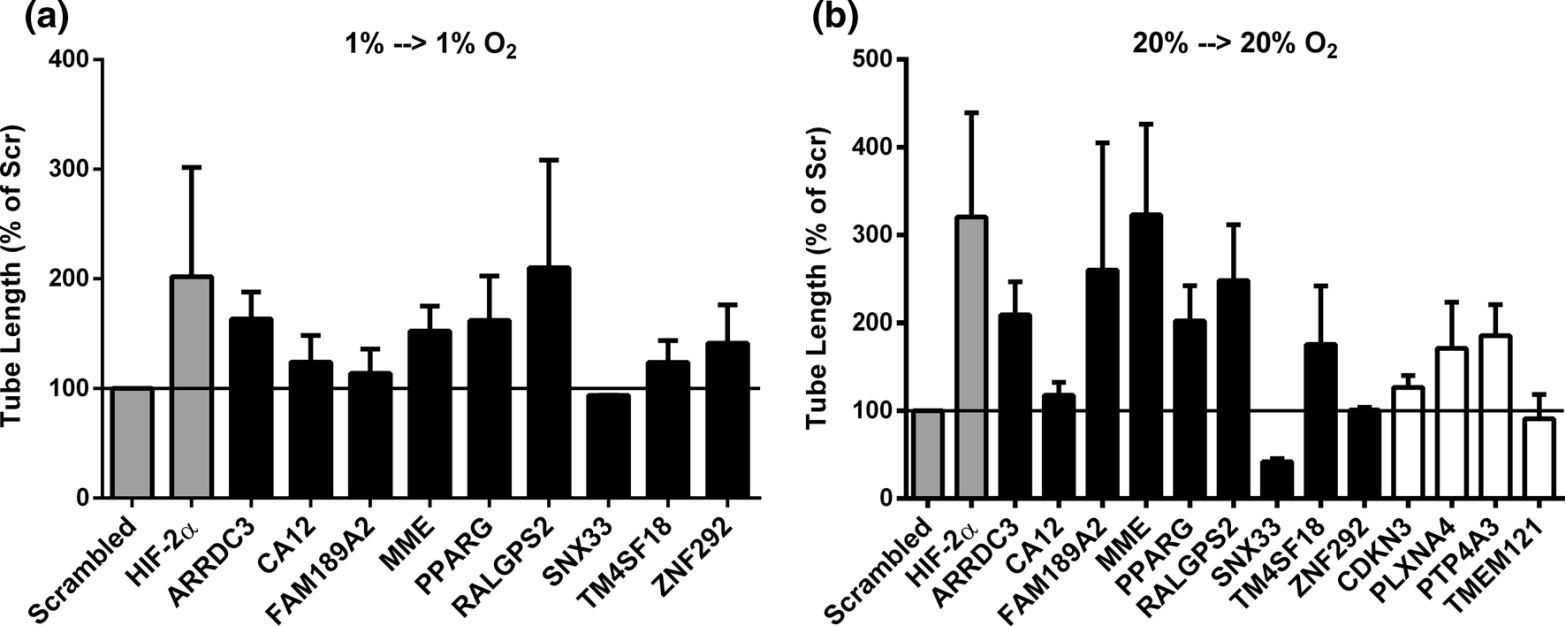
Effect of silencing candidate genes on endothelial sprouting in prolonged hypoxia and normoxia. hMVECs were precultured at 1 % oxygen **(a)** or 20 % oxygen **(b)** and silenced with one of the 13 genes selected from the screening. hMVECs were seeded on top of fibrin matrices before transfection with si-RNA. Subsequently, the hMVECs were stimulated with the combination of VEGF-A and TNFα. Tube length of hMVECs 7 days after stimulation with VEGF-A/TNFα was quantified by using Optimas software and expressed percentage of Scrambled with SEM (*n* = 3 independent donors, each in triplicate). The genes that were upregulated in prolonged hypoxia are expressed as black bars, and the genes that were downregulated in prolonged hypoxia are expressed as white bars. For statistical analysis, one-way ANOVA with Bonferroni post hoc test was used

Furthermore, we examined whether silencing of these genes may restore sprouting in hMVECs in prolonged hypoxia. By using a similar threshold of 25 % more sprouting compared to the scrambled control, we determined that silencing of ARRDC3, MME, PPARG and RALGPS2 partially restored sprouting in prolonged hypoxia (Fig. 5a). Finally, we investigated whether these genes also regulated endothelial sprouting under normoxic conditions. Silencing of ARRDC3, CDKN3, FAM189A2, MME, PLXNA4, PPARG, PTP4A3, RALGPS2 and TM4SF18 resulted in an increased sprouting of more than 25 % compared with scrambled, and silencing of SNX33 resulted in a decreased sprouting of more than 25 % compared with scrambled (Fig. 5b). Taken together, from the 13 candidate genes, silencing of ARRDC3, MME, PPARG and RALGPS2 increases sprouting more that 25 % compared with scrambled in all conditions.

## Discussion

This study identified HIF-2α-regulated genes that were involved in endothelial sprouting during hypoxia and normoxia and stimulated with VEGF-A/TNFα. Using genome-wide RNA-sequencing, we found that only 51 genes out of the ~12,000 known genes that were expressed in endothelial cells had an absolute fold change of >1.5 and were significantly regulated (FDR < 5 %) by both hypoxia and after HIF-2α silencing. Of these 51 genes, 13 candidate genes (ARRDC3, CA12, CDKN3, FAM189A2, MME, PLXNA4, PPARG, PTP4A3, RALGPS2, SNX33, TM4SF18, TMEM121, ZNF292) showed to regulate endothelial sprouting in short-term and long-term hypoxia and normoxia. Further examination showed that silencing of ARRDC3, MME, PPARG and RALGPS2 partly restored the prolonged hypoxia-induced inhibition of endothelial sprouting.

### Identifying oxygen- and HIF-2α-regulated genes involved in sprouting during prolonged hypoxia

The RNA-sequencing showed that VEGF-A was 3.5-fold induced during prolonged hypoxia. Although an increased VEGF-A expression in response to hypoxia was published before [4–6, 22–24, 29, 39], these studies only investigated short-term hypoxia (16–48 h). Despite the increased VEGF-A expression during prolonged hypoxia and the stimulation with VEGF-A and TNFα during the sprouting assay, the hMVECs were unable to form sprouts in prolonged hypoxia (Fig. 1). Moreover, the effects of prolonged hypoxia on VEGF receptors such as FLT1, KDR, NRP1, NRP2 were small (<15 % reduction or induction), which is probably caused by the presence of exogenous VEGF-A (10 ng/mL) in both normoxia and hypoxia. Both the increased VEGF-A expression and addition of excess exogenous VEGF-A exclude that limited VEGF-A availability causes reduced sprouting in prolonged hypoxia. Therefore, hypoxia-responsive genes upon VEGF-A/TNFα stimulation were explored in more detail.

The RNA-sequencing approach revealed 501 genes that were significantly upregulated in response to prolonged hypoxia. The top 10 of the genes with largest upregulation include EGLN3, HIF3A and SLC2A1. Upregulation of these genes in response to short-term hypoxia (16–72 h) was also observed in microarrays using human endothelial cells [4, 29] or tumor cells lines [22–24, 39]. In addition, we identified 333 genes that were significantly downregulated in response to prolonged hypoxia. The top 10 of the genes with largest downregulation include RRM2, MYBL2 and HMOX1, which is in line with the short-term hypoxia microarray studies [29].

Through silencing of HIF-2α with specific si-RNA, the prolonged hypoxia-induced inhibition of endothelial cells was partially restored. HIF-2α has often been studied in human tumor cell lines and human cells without functional HIF-1α [22–24, 27, 40, 41]. However, studies investigating gene regulation of HIF-2α in human endothelial cells with functional HIF-1α are absent. Our study examined HIF-2α-target genes by silencing of HIF-2α with si-RNA in prolonged hypoxia, which had no effect on HIF-1α expression. Upon silencing of HIF-2α, 449 genes were significantly upregulated and 715 were significantly downregulated compared with the gene expression in normoxia. Genes that were significantly regulated upon transfection with scrambled si-RNA in prolonged hypoxia were excluded from further analysis. Among the HIF-2α-regulated genes are LOXL2, RAB42 and MMP17, which have been identified as HIF-2α-target genes in short-term hypoxia [24, 27, 41].

It should be mentioned that—because of the transient nature of si-RNA transfection—si-RNA was added after the initial 2-week hypoxic preincubation at the onset of the 1-week evaluation of sprouting. Repeated transfections were avoided to limit cell damage or unwanted activation. Notwithstanding this limitation, our data clearly show partial rescue of prolonged hypoxia-induced inhibition of endothelial tube formation, but an underestimation of the effect of si-HIF-2α cannot be excluded yet.

### Metabolic pathways are increased in hypoxia and decreased by si-HIF-2α

KEGG pathway analysis revealed that the upregulated genes in long-term hypoxia were enriched in several metabolic pathways, such as glycolysis/gluconeogenesis, carbon-, fructose- and mannose metabolism (Table 2). During hypoxia, lack of oxygen prevents optimal functioning of the electron transport chain. However, inhibition of mitochondrial ATP production has very little effect on the endothelial cellular ATP level [42] and did not affect endothelial sprouting at normoxic conditions [43] (our own unpublished observations). As energy is mainly produced during glycolysis, it is functional that the glycolysis pathway is upregulated during hypoxia [28, 29, 44, 45]. It has been suggested that glycolysis genes are dependent on HIF-1 and not on HIF-2 [22]. Many of metabolic genes were significantly downregulated in our HIF-2α-depleted hMVECs (Table 4). Although the HIF-2α-regulated metabolic pathways include fatty acid- and carbon metabolism, glycolysis genes are not significantly altered by silencing of HIF-2α.

### Cell cycle pathway is decreased in hypoxia and increased upon si-HIF-2α

In contrast to the upregulated genes, the downregulated genes in long-term hypoxia were enriched within cell cycle and DNA replication pathways. This would suggest that endothelial cell proliferation was decreased in hypoxia. Although it has been shown that genes involved in cell cycle, DNA replication and DNA repair were also downregulated genes in response to short-term hypoxia in endothelial [29, 44] or tumor cells [4], we did not find significant differences in proliferation rates in hMVECs precultured for 14 days in hypoxia or normoxia [20].

To our surprise, many cell cycle genes were upregulated in HIF-2α-depleted hMVECs, suggesting an increased proliferative capacity of the hMVECs. However, under our experimental conditions, the hMVECs are stimulated with TNFα, which is shown to inhibit proliferation in endothelial cells [21]. Notwithstanding, the pathway analysis indicated that hypoxia, through HIF-2α, induced metabolic reprogramming and preservation of energy through decreased cell cycle and DNA replication.

### Are ARRDC3, PPARγ, RALGPS2, and MME involved in endothelial sprouting?

Combining the data of the oxygen- and HIF-2α-regulated genes, only 51 genes overlapped in opposite direction. These include a few known hypoxia-responsive genes [24], such as BNIP3L, LOXL2, PPARG and P4HA1. Only LOXL2 was described in the literature to be a HIF-2α-regulated gene [24]. However, further screening of the role of LOXL2 in endothelial sprouting showed that LOXL2 did not affect endothelial sprouting in hypoxia (Table 5). The tube formation screening revealed 13 genes that influenced sprouting during short-term hypoxia, but only 4 of these 13 genes (ARRDC3, MME, PPARγ and RALGPS2) also regulated endothelial sprout formation during prolonged hypoxia (compare Fig. 4b and Fig. 5a).

It is suggested that arrestin domain containing 3 (ARRDC3), also identified as TLIMP, plays a role in membrane protein internalization, like most members of the arrestin family [46–48]. Its expression was found in several human cancer cell lines and multiple human tissues, and not in endothelial cells, but is—surprisingly—induced by PPARγ [46]. In mammary tumor cells, repression of ARRDC3 enhanced the proliferation and migration [49].

Peroxisome proliferator-activated receptor gamma (PPARG) is expressed in many tissues with highest expression in adipose tissue and is mainly involved in lipid and glucose metabolism [50]. PPARG is also expressed in endothelial cells and is upregulated in response to short-term hypoxia (24–48 h) [4, 29]. Stimulation of PPARγ by agonists inhibited bFGF- and VEGF-stimulated angiogenesis, endothelial cell migration and proliferation [51–53]. Moreover, these PPARγ agonists inhibited tumor cell proliferation and angiogenesis in vivo [52]. However, in the lungs, loss of PPARγ results in decreased angiogenesis [54, 55].

Ral GEF with PH domain and SH3 binding motif 2 or RalA exchange factor (RALGPS2) is a member of the Ral GEF family of proteins. Unlike most Ral GEF proteins, RALGPS2 has a Ras-independent function and is probably involved in actin polymerization and cytoskeleton organization through binding to actin filaments [56]. RALGPS2 has highest expression in the testis or brain [56, 57]. It is suggested that RALGPS2 functions as an inhibitor of RalA signaling and thereby decreases tumor cell proliferation and induces apoptosis [58, 59]. The role of RALGPS2 in endothelial cells is probably different, because no cell death was observed during our 7-day tube formation assay.

It is known that membrane metalloendopeptidase (MME) plays a role during angiogenesis [60, 61]. MME, also known as neutral endopeptidase or neprilysin (NEP), CD10 or common acute lymphoblastic leukemia antigen (CALLA), is a zinc-dependent metalloprotease enzyme involved in the cleavage and inactivation of certain peptide hormones involved in signal transduction [61, 62]. Its expression has been found in many tumor cells [63] and several tissues including epithelial and endothelial cells [64, 65]. mRNA expression was increased in lung cancer cells and lung fibroblasts in response to hypoxia [28], but decreased in human pulmonary arterial smooth muscle cells, murine lungs [66] or murine lung and renal homogenates [67]. Moreover, MME inhibits endothelial FGF-2-stimulated angiogenesis, proliferation and migration [60, 61]. In our study, we showed that MME also inhibits VEGF-stimulated angiogenesis.

## Conclusion

In conclusion, this study identified four novel HIF-2α target genes that inhibit endothelial sprouting during prolonged hypoxia in vitro. Of these four genes, PPARγ and MME have previously been linked to angiogenesis, while ARRDC3 and RALGPS2 only have been shown to influence cell proliferation. PPARG is an interesting gene for further investigation; it induces the expression of ARRDC3 and plays a role in both angiogenesis and metabolism. In particular, the metabolism pathway was enriched among the upregulated genes in response to hypoxia. This suggests that PPARγ and ARRDC3 are potential targets to restore reduced neovascularization in several pathological conditions, such as diabetic or other chronic ischemic wounds, or for improvement of vascularization of implanted tissue-engineered scaffolds.

## Electronic supplementary material

Below is the link to the electronic supplementary material.

**Supplementary Table** **1. Primer sequences for qRT-PCR.** (PDF 102 kb)

**Supplementary Table** **2. All genes with significantly induced or repressed gene expression in prolonged hypoxic precultured hMVECs after VEGF-A/TNF-α stimulation.** The relative gene expression was compared with the gene expression of hMVECs precultured in normoxia and stimulated with VEGF-A/TNF-α (n = 4 independent donors). (PDF 486 kb)

**Supplementary Table** **3. Gene expression data from RNA-sequencing; the HIFα subunits.** The relative gene expression in prolonged hypoxia was compared with the gene expression in normoxia, the gene expression with si-HIF-2α and scrambled was compared with hypoxia (untransfected) upon VEGF-A/TNF-α stimulation (n = 4 independent donors). (PDF 202 kb)

**Supplementary Table** **4. All genes with significantly induced or repressed gene expression upon HIF-2α knock-down in prolonged hypoxic precultured hMVECs after VEGF-A/TNF-α stimulation.** The relative gene expression was compared with the gene expression of hMVEC precultured in hypoxia and stimulated with VEGF-A/TNF-α (n = 4 independent donors). (PDF 530 kb)
