# Supplementary material for: Identification of HIF-2α-regulated genes that play a role in human microvascular endothelial sprouting during prolonged hypoxia in vitro
Source: Angiogenesis. 2016 Oct 3;20(1):39–54. doi: 10.1007/s10456-016-9527-4 (PMC5306362; doi:10.1007/s10456-016-9527-4)
Supplement: Supplementary file 1 — Supplementary Table 1. Primer sequences for qRT-PCR. (PDF 102 kb) [file 10456_2016_9527_MOESM1_ESM.pdf]

Supplementary Table 1. Primer sequences for qRT-PCR

Gene Symbol	Forward Primer	Reverse Primer
**B2M [1]**	TTTCATCCATCCGACATTG	CGGCAGGCATACTCATCTTT
**ARRDC3**	GCGAAAGTACGCTGGACTGA	TGAGGTAGCGAGTGGTGTCT
**CA12**	ACTTATTTTGGTCCTGATGGGG	TGGCCATTGTTGGTCAGGAG
**CDKN3**	CTTGTCTTGTAGCTGCTTGTCTC	TGCAGCTAATTTGTCCCGAAAC
**FAM189A2** [2]	CTTGAGGACCAGGTCGAAGAGT	CTCCTTTCAGTCTGTGTTGCAGC
**MME** [2]	CTTTAGTGCCCAGCAGTCCAAC	CACCAGTCAACGAGGTCTCCAT
**PLXNA4** [2]	TCGTGCGGATTGAGCCAGAATG	TGATGTGCTCCTTCCCTCCATG
**PPARG**	CGACCAGCTGAATCCAGAGT	GATGCGGATGGCCACCTCTT
**PTP4A3**	ACAAGCACAGGGATCTCGTT	CTTCAGGTCCTCAATGAAGGTG
**RALGPS2**	GCAGTCAGTCCTCTGTTGCT	TCTGACCCGCATATTCTTCTGG
**SNX33**	GCTTTGTGCGTTCTGGAGTG	TGGTCTGTTTTGTGGGGTCC
**TM4SF18**	ATGTTGCCAGAGTGAAAACTGC	CTGTAAGGAAACGTCCAGCA
**TMEM121** [2]	AGCACATAGCGCCGCAGAAGAT	CGTTTTTGCCGACGAAGATGGC
**ZNF292**	GTGTGAAGATGGCGGACGAA	GCATATTCTAGGAGTGTCTGGCA

References

1. Weijers EM, van Wijhe MH, Joosten L, et al. (2010) Molecular weight fibrinogen variants alter gene expression and functional characteristics of human endothelial cells. J Thromb Haemost 8:2800–9. doi: 10.1111/j.1538-7836.2010.04096.x

2. OriGene. http://www.origene.com/. Accessed 29 Jan 2016
