# Supplementary material for: Identification of HIF-2α-regulated genes that play a role in human microvascular endothelial sprouting during prolonged hypoxia in vitro
Source: Angiogenesis. 2016 Oct 3;20(1):39–54. doi: 10.1007/s10456-016-9527-4 (PMC5306362; doi:10.1007/s10456-016-9527-4)
Supplement: Supplementary file 2 — Supplementary Table 2. All genes with significantly induced or repressed gene expression in prolonged hypoxic precultured hMVECs after VEGF-A/TNF-α stimulation. The relative gene expression was compared with the gene expression of hMVECs precultured in normoxia and stimulated with VEGF-A/TNF-α (n = 4 independent donors). (PDF 486 kb) [file 10456_2016_9527_MOESM2_ESM.pdf]

**Supplementary Table 2. All genes with significantly induced or repressed gene expression in prolonged hypoxic precultured hMVECs after VEGF-A/TNF-α stimulation.**

| Upregulated in Hypoxia | | Downregulated in Hypoxia | |
|---|---|---|---|
| Gene | Fold Change | Gene | Fold Change |
| ABCC3 | 2.1 | A1BG,ZNF497 | 0.55 |
| ABTB1 | 1.5 | A1BG-AS1 | 0.53 |
| ACCN3 | 1.9 | AATK | 0.64 |
| ACER2 | 1.7 | ABCA6 | 0.52 |
| ACTR3C | 4.1 | ACPL2 | 0.46 |
| ADAMTS3 | 1.7 | ACTR3B | 0.66 |
| ADAMTSL3 | 1.6 | ADAM19 | 0.65 |
| ADM | 3.5 | ADAMTS12 | 0.38 |
| ADM2 | 2.4 | ADAMTS18 | 0.46 |
| ADPRH | 1.8 | ADAT2 | 0.64 |
| ADRB1 | 2.3 | ADC | 0.44 |
| ADSSL1 | 2.8 | AGAP11,C10orf116 | 0.51 |
| AEBP1,MIR4649 | 2.9 | AGPAT9 | 0.57 |
| AGMO | 2.8 | ALDH1A3 | 0.63 |
| AIM1 | 1.7 | ALDH4A1,MIR4695 | 0.53 |
| AJUBA | 1.9 | AMD1 | 0.66 |
| AK4 | 2.8 | ANKRD23,ANKRD39 | 0.58 |
| ALDOA | 1.6 | ANKRD55 | 0.48 |
| ALDOC | 2.2 | ANLN | 0.45 |
| ALPK3,ZNF592 | 1.5 | ANXA6 | 0.63 |
| AMIGO2 | 2.5 | ARHGAP11A,SCG5 | 0.52 |
| ANGPTL4 | 4.0 | ARHGAP5-AS1 | 0.66 |
| ANK1 | 2.1 | ARMCX2 | 0.67 |
| ANKRD37 | 7.4 | ASF1B | 0.35 |
| ANKRD53 | 2.6 | ATAD3A,ATAD3B | 0.66 |
| ANO2 | 2.0 | ATF7IP2 | 0.67 |
| ANTXR2 | 1.5 | ATP2B4 | 0.66 |
| ANXA3 | 1.8 | ATP6V0E2 | 0.43 |
| ARHGAP21 | 1.6 | AURKA | 0.57 |
| ARHGAP4 | 1.7 | AURKB | 0.26 |
| ARHGEF40 | 1.8 | B9D1 | 0.64 |
| ARID5A | 1.7 | BCAN | 0.48 |
| ARRDC3 | 4.3 | BHLHE41 | 0.51 |
| ARRDC4 | 3.8 | BIRC5 | 0.31 |
| ATF3 | 2.0 | BRCA1 | 0.44 |
| B3GALT4 | 2.0 | BRI3BP | 0.66 |
| BACH1 | 3.3 | BTBD11 | 0.36 |
| BAIAP2L1 | 1.5 | BUB1 | 0.31 |

| | | | |
|---|---|---|---|
| BATF | 1.9 | BUB1B,PAK6 | 0.35 |
| BATF3,NSL1 | 1.6 | C10orf125 | 0.65 |
| BCAR1 | 1.7 | C11orf96 | 0.53 |
| BCL6 | 1.5 | C16orf59 | 0.56 |
| BEST1 | 1.9 | C17orf28 | 0.61 |
| BEST4,PTCH2,TCTEX1D4 | 1.8 | C1orf233 | 0.60 |
| BEX5 | 1.8 | C20orf27 | 0.61 |
| BGN | 2.6 | C2CD4A | 0.44 |
| BHLHE40 | 1.9 | C6orf97 | 0.55 |
| BHMT2 | 2.8 | CADM4 | 0.56 |
| BMF | 1.7 | CAMK1 | 0.64 |
| BMP4 | 2.3 | CAMK2N1 | 0.66 |
| BMPER | 3.0 | CBLN2 | 0.55 |
| BNIP3 | 1.6 | CBX6 | 0.64 |
| BNIP3L | 2.0 | CCDC102B | 0.56 |
| BTG1,LOC256021 | 1.7 | CCDC147 | 0.61 |
| C10orf10 | 1.5 | CCDC165 | 0.63 |
| C11orf35 | 1.8 | CCDC169,CCDC169-SOHLH2,SOHLH2 | 0.46 |
| C12orf39 | 2.0 | CCNB2,MYO1E | 0.55 |
| C15orf48,MIR147B | 2.4 | CCND1 | 0.65 |
| C1orf133 | 6.9 | CCNE1 | 0.39 |
| C1orf21 | 1.8 | CCNE2 | 0.49 |
| C1QTNF1 | 2.9 | CD34 | 0.58 |
| C1R | 1.6 | CDC20 | 0.36 |
| C20orf96 | 1.5 | CDC45 | 0.19 |
| C21orf7 | 1.9 | CDCA2 | 0.28 |
| C3orf15 | 1.6 | CDCA5,LOC100130348 | 0.59 |
| C4orf3 | 1.7 | CDCA8 | 0.37 |
| C4orf47 | 3.8 | CDK1 | 0.24 |
| C6orf132 | 2.3 | CDKN2A | 0.32 |
| C7orf29,LRRC61 | 3.2 | CDKN3 | 0.64 |
| C7orf31 | 1.8 | CENPA | 0.31 |
| C7orf58 | 2.2 | CENPM | 0.38 |
| C7orf61 | 1.9 | CEP55 | 0.28 |
| C7orf63 | 1.5 | CES4A | 0.64 |
| C8orf4 | 1.6 | CGNL1 | 0.63 |
| C8orf58 | 1.7 | CHAC2 | 0.65 |
| C9orf5 | 2.3 | CHAF1B | 0.56 |
| CA11 | 1.7 | CHCHD10 | 0.66 |
| CA12 | 2.0 | CHN2 | 0.47 |
| CA2 | 6.5 | CHTF18 | 0.64 |
| CABP4 | 1.6 | CIT,MIR1178 | 0.44 |

| | | | |
|---|---|---|---|
| CACNA1A | 1.6 | CKAP2L | 0.40 |
| CACNA1C | 2.2 | CLEC7A | 0.54 |
| CAMK1D,LOC283070 | 2.2 | CLSPN | 0.35 |
| CAPN12 | 2.1 | CORO1A,LOC606724 | 0.51 |
| CARHSP1 | 1.5 | CORO2B | 0.57 |
| CBS | 1.6 | CPE | 0.47 |
| CCL20 | 1.8 | CRHR1,MGC57346 | 0.67 |
| CCL28 | 2.2 | CXCL6 | 0.64 |
| CCNG2 | 1.5 | CXorf36 | 0.18 |
| CCR10 | 1.5 | CYP2S1 | 0.24 |
| CCRN4L | 1.6 | CYTL1 | 0.57 |
| CD101 | 1.5 | DCBLD2 | 0.65 |
| CD302,LY75,LY75-CD302 | 1.8 | DGAT2 | 0.61 |
| CD69 | 2.3 | DGKG | 0.54 |
| CDCP1 | 2.0 | DHRS11 | 0.49 |
| CDH2 | 2.9 | DLGAP5 | 0.28 |
| CEBPD | 1.6 | DLL1 | 0.53 |
| CELSR1 | 1.6 | DNAJB4 | 0.54 |
| CEP112 | 1.6 | DNAJC6 | 0.61 |
| CEP135 | 1.7 | DPY19L3 | 0.62 |
| CHAC1 | 2.4 | DPYSL4 | 0.66 |
| CHRNB1 | 1.5 | EDNRB | 0.62 |
| CHRNE | 1.9 | EFNB2 | 0.64 |
| CHST2 | 1.6 | ELOVL6 | 0.51 |
| CHST6 | 1.9 | EME1 | 0.36 |
| CHSY3 | 1.8 | EPHX1 | 0.55 |
| CLCN4 | 1.7 | ERCC2 | 0.66 |
| CLDN14 | 2.3 | FAM101B | 0.67 |
| CLDN23 | 2.0 | FAM102A | 0.63 |
| CLDN7 | 1.7 | FAM109A | 0.61 |
| CLEC2B | 1.8 | FAM117A | 0.65 |
| CMPK2 | 1.6 | FAM171A2 | 0.56 |
| COL12A1 | 2.7 | FAM212B | 0.53 |
| COL1A2 | 6.0 | FAM216A | 0.61 |
| COL8A2 | 2.9 | FAM54A | 0.58 |
| CPLX1 | 3.1 | FAM59B | 0.52 |
| CRNDE | 1.5 | FAM83D | 0.25 |
| CSPG4 | 2.9 | FANCA | 0.54 |
| CSPG5 | 2.9 | FANCD2 | 0.48 |
| CSRP2 | 2.0 | FANK1 | 0.60 |
| CTF1 | 1.8 | FEN1 | 0.64 |
| CXCR7 | 1.9 | FKBP14 | 0.65 |
| CYP1A1 | 2.0 | FLJ42627 | 0.56 |

| | | | |
|---|---|---|---|
| CYP27A1,TTLL4 | 1.9 | FN3K | 0.67 |
| CYP2J2 | 1.6 | FRMD5 | 0.60 |
| DACT1 | 4.5 | GADL1 | 0.36 |
| DAPK2 | 1.6 | GAMT | 0.55 |
| DARS | 1.6 | GCAT | 0.47 |
| DCHS1 | 1.6 | GFOD1 | 0.57 |
| DDIT3 | 1.7 | GINS1 | 0.55 |
| DDIT4 | 3.6 | GINS2 | 0.21 |
| DDX43 | 2.5 | GMFB | 0.61 |
| DNAH5 | 1.7 | GREB1L | 0.63 |
| DNAJC18 | 1.6 | GTF2IP1,LOC100093631 | 0.59 |
| DNMT3A | 1.5 | GTSE1 | 0.32 |
| DOCK2 | 1.6 | HBEGF | 0.56 |
| DTX4 | 1.8 | HCN2 | 0.57 |
| DUSP5P,RHOU | 1.6 | HES7 | 0.53 |
| DYRK2 | 1.8 | HEY2 | 0.64 |
| EBF4 | 3.5 | HIF1A | 0.53 |
| EBI3 | 1.8 | HJURP | 0.38 |
| EDIL3 | 1.5 | HMMR | 0.41 |
| EFNA1 | 1.7 | HMOX1 | 0.19 |
| EGLN1 | 1.7 | HS3ST1 | 0.55 |
| EGLN3 | 106.8 | IFI27L1 | 0.66 |
| ENKUR | 1.7 | IFI27L2 | 0.66 |
| ENO2 | 3.1 | IGF1 | 0.16 |
| ENO3 | 1.6 | IL6R | 0.61 |
| EPB41L3 | 1.7 | IPW,SNORD116-17,SNORD116-21,SNORD116-24,SNORD116-26,SNORD116-4 | 0.67 |
| EPSTI1 | 1.5 | JAG1 | 0.66 |
| ERO1LB | 1.6 | KIAA0513 | 0.63 |
| ERRFI1 | 3.8 | KIAA1274 | 0.36 |
| F2RL3 | 5.8 | KIF11 | 0.60 |
| FAIM | 1.5 | KIF19 | 0.36 |
| FAM102B | 1.5 | KIF20B | 0.58 |
| FAM115C | 2.5 | KIF22 | 0.66 |
| FAM117B | 1.8 | KIF23 | 0.57 |
| FAM13A | 3.7 | KIF2C | 0.33 |
| FAM150B | 3.9 | KIF4A | 0.39 |
| FAM160A1 | 1.9 | KIFC1 | 0.35 |
| FAM167B | 1.6 | KLHL4 | 0.67 |
| FAM189A2 | 1.6 | KYNU | 0.07 |
| FAM26F | 3.0 | L2HGDH | 0.50 |

| | | | |
|---|---|---|---|
| FAM46A | 1.9 | L3MBTL3 | 0.62 |
| FAM46C | 1.9 | LAMA3 | 0.60 |
| FBLN7 | 2.1 | LAPTM4B | 0.61 |
| FER1L4 | 2.3 | LDB2 | 0.57 |
| FLJ13197 | 1.9 | LDLRAD2 | 0.59 |
| FLJ32224 | 2.7 | LINC00520 | 0.47 |
| FLJ34503 | 2.0 | LOC100507053 | 0.61 |
| FLJ44511 | 1.5 | LOC100507254 | 0.35 |
| FOXS1 | 2.0 | LOC284751 | 0.62 |
| FST | 3.1 | LOC285758 | 0.65 |
| FXYD2,FXYD6,FXYD6-FXYD2 | 1.9 | LOC388849 | 0.66 |
| FZD8 | 1.7 | LOC401052 | 0.43 |
| GABRE,MIR452 | 1.7 | LOC728084 | 0.40 |
| GADD45B | 2.1 | LRP3 | 0.63 |
| GAL3ST4 | 3.5 | LRRC20 | 0.36 |
| GALM | 2.0 | LRRC33 | 0.50 |
| GALNTL4 | 2.3 | LRRC8B | 0.51 |
| GATA6 | 1.6 | LST1 | 0.44 |
| GCNT4 | 1.9 | MAPKAPK3 | 0.62 |
| GEM | 2.2 | MAPRE3 | 0.63 |
| GHDC | 2.1 | MARCKSL1 | 0.64 |
| GHRL | 1.8 | MCM4 | 0.48 |
| GIMAP1,GIMAP1-GIMAP5,GIMAP5 | 2.1 | MEG3 | 0.66 |
| GJC2 | 1.9 | MEX3A | 0.52 |
| GLIS3 | 1.7 | MFSD2A | 0.23 |
| GNA14 | 3.2 | MGC23284 | 0.66 |
| GNAL | 2.4 | MGP | 0.35 |
| GPR146 | 2.5 | MIR31HG | 0.66 |
| GPR68 | 1.7 | MKI67 | 0.18 |
| GPX7 | 2.0 | MLF1IP | 0.45 |
| GREM1 | 2.7 | MMP15 | 0.51 |
| GULP1 | 1.8 | MMP16 | 0.58 |
| GZMA | 1.7 | MRAS | 0.52 |
| H1F0 | 1.9 | MREG,PECR | 0.58 |
| H1FX | 1.5 | MXRA8 | 0.59 |
| HAVCR2 | 2.6 | MYBL2 | 0.13 |
| HELB | 1.6 | MYRIP | 0.59 |
| HERC5 | 2.1 | NCAPG | 0.30 |
| HHIPL1 | 2.0 | NDC80 | 0.22 |
| HIF3A | 11.1 | NEU3 | 0.65 |
| HILPDA | 1.8 | NID2 | 0.27 |

| | | | |
|---|---|---|---|
| HIST1H2BD | 1.5 | NLGN1 | 0.34 |
| HIST2H2BE | 1.7 | NOVA1 | 0.56 |
| HK2 | 2.0 | NPM3 | 0.58 |
| HLA-A | 1.8 | NRGN | 0.66 |
| HOXD1 | 1.8 | NUF2 | 0.27 |
| HOXD-AS1 | 1.5 | NUSAP1 | 0.45 |
| HRH1 | 1.6 | OR5B17 | 0.63 |
| HSD17B2 | 1.6 | ORC6 | 0.46 |
| HSPA12B | 2.8 | OSBP2 | 0.59 |
| HTR1B | 1.8 | OTUB2 | 0.62 |
| HVCN1 | 2.0 | PANK1 | 0.63 |
| HYAL4 | 3.8 | PARD6G | 0.39 |
| ICAM4 | 2.9 | PARPBP | 0.59 |
| ICAM5 | 2.2 | PASK | 0.62 |
| IFITM1 | 1.8 | PDXP | 0.64 |
| IFITM10 | 1.9 | PFN2 | 0.61 |
| IGFBP5 | 7.0 | PHEX | 0.60 |
| IL18R1 | 1.6 | PIGZ | 0.53 |
| IL1R1 | 1.8 | PIK3R3 | 0.45 |
| IL1RAP | 2.1 | PKMYT1 | 0.23 |
| IL2RG | 1.5 | PLA2G4A | 0.54 |
| IL4I1,NUP62 | 1.5 | PLXNA4 | 0.37 |
| IL6 | 2.0 | PMCH | 0.37 |
| INHBA | 2.1 | PODXL2 | 0.44 |
| IPMK | 1.5 | POLR3G | 0.57 |
| IRF7 | 1.6 | POP1 | 0.47 |
| IRS2 | 1.7 | PPARGC1B | 0.63 |
| ISL2 | 1.7 | PPP1R16B | 0.66 |
| ITGB4 | 1.8 | PRDX2 | 0.67 |
| ITGBL1 | 2.3 | PRR7 | 0.30 |
| JDP2 | 2.0 | PRRT3 | 0.54 |
| JPH2 | 2.5 | PRRX1 | 0.29 |
| KANK3 | 1.6 | PRSS3 | 0.62 |
| KCNJ2 | 1.8 | PSRC1 | 0.51 |
| KCNS3 | 4.2 | PTGDS | 0.49 |
| KCTD16 | 4.9 | PTP4A3 | 0.40 |
| KCTD8 | 4.1 | PTPRS | 0.37 |
| KDM3A | 1.9 | PTTG3P | 0.58 |
| KLF11 | 1.6 | PVRL1 | 0.67 |
| KLF4 | 3.5 | PXMP2 | 0.54 |
| KLHL3,MYOT | 1.7 | QPCTL | 0.62 |
| L3MBTL4 | 1.9 | RAB3A | 0.61 |
| LAG3 | 1.9 | RAB6B | 0.29 |

| | | | |
|---|---|---|---|
| LDHA | 2.2 | RAC3 | 0.57 |
| LIMCH1 | 4.7 | RACGAP1 | 0.58 |
| LIMS3,LIMS3-LOC440895,LOC100288570,LOC440895 | 1.8 | RAD51 | 0.63 |
| LINC00263 | 1.8 | RAD51AP1 | 0.37 |
| LINC00324 | 3.0 | RCAN2 | 0.32 |
| LITAF | 2.4 | RECQL4 | 0.52 |
| LOC100127983 | 1.9 | REEP6 | 0.66 |
| LOC100128071 | 1.8 | RFC3 | 0.57 |
| LOC100129726 | 2.0 | RGS10 | 0.61 |
| LOC100132077 | 2.9 | RGS11 | 0.43 |
| LOC100132891 | 2.0 | RGS4 | 0.42 |
| LOC100133445,LOC115110 | 1.8 | RGS5 | 0.15 |
| LOC100134259 | 2.5 | RHOBTB3 | 0.63 |
| LOC100190938 | 2.6 | RHPN1 | 0.63 |
| LOC100506178 | 1.7 | RPS6KL1 | 0.58 |
| LOC100506746 | 1.9 | RRM2 | 0.11 |
| LOC100507050 | 1.9 | SCAMP5 | 0.43 |
| LOC149837 | 2.6 | SCARB1 | 0.56 |
| LOC154761 | 2.6 | SCN5A | 0.52 |
| LOC283104 | 1.6 | SDC1 | 0.37 |
| LOC284454,MIR23A,MIR24-2,MIR27A | 1.7 | SDSL | 0.60 |
| LOC439949 | 1.8 | SFMBT1 | 0.55 |
| LOC440028 | 3.0 | SFXN2 | 0.50 |
| LOX | 2.0 | SH3PXD2A | 0.57 |
| LOXL2 | 1.5 | SH3TC2 | 0.49 |
| LRG1 | 1.6 | SHANK1 | 0.46 |
| LRP2BP | 1.7 | SHMT1,TOP3A | 0.49 |
| LRRC34 | 1.8 | SKA1 | 0.24 |
| LRRC49 | 1.6 | SLAIN1 | 0.64 |
| LRRC56 | 1.5 | SLC12A2 | 0.61 |
| LURAP1L | 1.6 | SLC25A10 | 0.51 |
| LYPD1 | 2.0 | SLC25A19 | 0.65 |
| LYPD5 | 2.1 | SLC25A23 | 0.55 |
| MAF | 2.2 | SLC25A29 | 0.61 |
| MAFK | 1.6 | SLC29A4 | 0.63 |
| MAMDC4 | 1.8 | SLC2A11 | 0.65 |
| MAP3K8 | 1.9 | SLC35G1 | 0.66 |
| MAPK13 | 2.2 | SLC40A1 | 0.48 |
| MB21D1 | 1.7 | SLC43A2 | 0.64 |
| MCHR1 | 3.2 | SLC44A5 | 0.39 |

| | | | |
|---|---|---|---|
| MEGF10 | 1.8 | SLC45A4 | 0.59 |
| MEGF6 | 1.9 | SLC47A1 | 0.38 |
| MEIS1 | 2.0 | SNRNP25 | 0.63 |
| MEX3B | 1.5 | SPAG5 | 0.48 |
| MGC16121,MIR424,MIR503, MIR542 | 3.1 | SPSB2 | 0.65 |
| MIR101-1,MIR3671 | 2.3 | SSBP3 | 0.60 |
| MIR146A | 1.7 | STEAP1B | 0.59 |
| MIR181A1,MIR181B1 | 2.4 | SULT1B1 | 0.41 |
| MIR210HG | 7.9 | TDRKH | 0.63 |
| MIR30B | 2.2 | TFRC | 0.64 |
| MLLT4-AS1 | 1.8 | TIMP1 | 0.61 |
| MME | 2.2 | TK1 | 0.32 |
| MMRN2 | 1.5 | TMEM106C | 0.55 |
| MOB3B | 1.6 | TMEM121 | 0.50 |
| MOXD1 | 1.6 | TMEM177 | 0.66 |
| MRGPRX3 | 2.5 | TMEM38A | 0.56 |
| MSC | 2.1 | TMEM38B | 0.67 |
| MT1F | 2.3 | TMEM97 | 0.53 |
| MT1L | 1.6 | TNFSF4 | 0.47 |
| MT1M | 5.9 | TNFSF9 | 0.52 |
| MUC1 | 1.9 | TNIK | 0.43 |
| MXI1 | 1.9 | TOP2A | 0.19 |
| MYCN | 2.6 | TPX2 | 0.54 |
| MYO15B | 2.8 | TRAP1 | 0.65 |
| NALCN | 1.6 | TRIML2 | 0.51 |
| NARF | 1.5 | TRIP13 | 0.52 |
| NDRG1 | 1.8 | TROAP | 0.43 |
| NDUFA4L2 | 1.6 | TUBA4A | 0.54 |
| NEURL3 | 2.0 | TYRO3 | 0.60 |
| NFIL3 | 1.7 | UBD | 0.50 |
| NKAPL | 1.9 | UBE2C | 0.44 |
| NKAPP1 | 1.6 | UBE2T | 0.49 |
| NLRC3 | 1.7 | UCHL1 | 0.63 |
| NOD2 | 1.7 | UCP2 | 0.53 |
| NOV | 3.0 | UHRF1 | 0.39 |
| NOX4 | 1.7 | VPS37D | 0.53 |
| NPR2 | 1.5 | VSIG1 | 0.67 |
| NR4A2 | 1.9 | WDHD1 | 0.61 |
| NTN1 | 1.9 | WDR62 | 0.44 |
| NTN4 | 1.6 | ZCWPW2 | 0.62 |
| NUPR1 | 1.7 | ZNF30 | 0.65 |
| NYNRIN | 1.7 | ZNF323 | 0.46 |

| | | | |
|---|---|---|---|
| OASL | 1.7 | ZNF485 | 0.60 |
| ODF3B | 2.6 | ZNF491 | 0.66 |
| OSMR | 2.4 | ZNF503 | 0.46 |
| OTUD1 | 1.6 | ZWINT | 0.54 |
| P2RX2 | 2.0 | | |
| P4HA1 | 2.0 | | |
| PAG1 | 3.2 | | |
| PALMD | 1.6 | | |
| PAN3-AS1 | 1.6 | | |
| PAPL | 2.0 | | |
| PAPPA2 | 2.3 | | |
| PCDH10 | 3.0 | | |
| PDE4C | 15.0 | | |
| PDGFRA | 1.8 | | |
| PDGFRL | 1.7 | | |
| PDK1 | 2.0 | | |
| PDZD2 | 1.9 | | |
| PGM1 | 2.5 | | |
| PGM5P2 | 1.6 | | |
| PHGDH | 2.5 | | |
| PIK3IP1 | 1.5 | | |
| PIM1 | 6.0 | | |
| PKP3 | 2.0 | | |
| PLA2G16 | 1.5 | | |
| PLCH2 | 2.7 | | |
| PLIN2 | 1.7 | | |
| PLOD2 | 2.0 | | |
| PMAIP1 | 1.9 | | |
| PMEPA1 | 1.7 | | |
| PNMA2 | 1.7 | | |
| PODXL | 1.9 | | |
| POF1B | 2.4 | | |
| POMC | 2.5 | | |
| PP7080,SLC9A3 | 1.8 | | |
| PPARG | 6.5 | | |
| PPFIA4 | 22.9 | | |
| PPP1R13L | 1.7 | | |
| PPP1R3G | 6.7 | | |
| PRDM1 | 1.7 | | |
| PRDM8 | 2.4 | | |
| PRKCQ | 2.7 | | |
| PRKY | 2.1 | | |
| PROCA1 | 1.9 | | |

| | |
|---|---|
| PROS1 | 1.6 |
| PRPS2 | 1.5 |
| PTER | 2.0 |
| PTGFR | 1.6 |
| PTGIR | 2.2 |
| PTPRB | 2.0 |
| QPCT | 2.0 |
| RAB33A | 2.6 |
| RAB34 | 1.7 |
| RAB38 | 1.8 |
| RALGPS2 | 1.9 |
| RAMP2 | 2.5 |
| RARRES1 | 2.9 |
| RASA4 | 1.5 |
| RASSF4 | 1.8 |
| RBMS3 | 1.5 |
| REPS2 | 2.1 |
| RGAG4 | 1.6 |
| RGNEF | 1.6 |
| RGS7 | 2.0 |
| RHBDF2 | 1.6 |
| RHEBL1 | 1.7 |
| RIPK4 | 3.8 |
| RLF | 1.6 |
| RNASEL | 1.6 |
| RORA | 1.8 |
| RPS6KA5 | 1.6 |
| RSPH9 | 1.9 |
| RTKN | 2.5 |
| S100A1 | 1.5 |
| SARM1 | 2.1 |
| SAV1 | 1.6 |
| SCARNA9 | 1.8 |
| SCO2,TYMP | 1.7 |
| SDC4 | 2.1 |
| SDR42E1 | 1.6 |
| SEMA3C | 1.8 |
| SEMA4B | 2.4 |
| SEPT4 | 3.2 |
| SERTAD4 | 7.0 |
| SFMBT2 | 1.8 |
| SGCE | 2.2 |
| SGIP1 | 1.9 |

| | |
|---|---|
| SH3D21 | 2.7 |
| SHH | 4.5 |
| SIPA1L1 | 1.9 |
| SIX1 | 2.7 |
| SIX4 | 1.9 |
| SLC16A3 | 1.9 |
| SLC2A1 | 7.3 |
| SLC2A3 | 2.7 |
| SLC30A1 | 1.5 |
| SLC38A4 | 1.9 |
| SLC39A14 | 1.6 |
| SLC45A1 | 1.7 |
| SLC6A6 | 1.6 |
| SLC6A9 | 2.8 |
| SLC7A2 | 2.0 |
| SLC7A5 | 2.0 |
| SLC8A1 | 2.2 |
| SLC8A3 | 11.3 |
| SLCO4A1 | 2.0 |
| SMAD7 | 3.3 |
| SMS | 1.5 |
| SNX33 | 1.6 |
| SP4 | 1.5 |
| SPAG4 | 2.1 |
| SPRY1 | 2.0 |
| SSTR2 | 1.7 |
| ST8SIA6 | 5.7 |
| STC2 | 3.2 |
| STMN3 | 1.7 |
| STX1B | 1.6 |
| SUN2 | 1.6 |
| SYNPO | 1.8 |
| TCHH | 2.3 |
| TCTEX1D1 | 4.0 |
| TESK2 | 1.7 |
| TGFB2 | 2.4 |
| TGFBI | 2.3 |
| TIMP3 | 3.0 |
| TIMP4 | 25.9 |
| TLE4 | 1.6 |
| TLR2 | 1.9 |
| TM4SF18 | 1.5 |
| TMEM173 | 1.7 |

| | |
|---|---|
| TMEM45A | 2.4 |
| TMEM53 | 1.5 |
| TMEM71 | 2.4 |
| TMIE | 2.0 |
| TNFAIP3 | 1.8 |
| TNFAIP6 | 1.7 |
| TNFRSF10B | 1.6 |
| TNFRSF10D | 1.7 |
| TNFRSF11A | 1.7 |
| TNFRSF4 | 1.5 |
| TOX | 2.3 |
| TP63 | 1.9 |
| TPI1 | 2.0 |
| TPM1 | 2.1 |
| TSPAN2 | 3.5 |
| TSPY26P | 1.7 |
| TTC30A | 1.7 |
| TTC30B | 1.8 |
| TUSC1 | 2.0 |
| TUSC3 | 1.5 |
| TXNIP | 3.9 |
| UBXN11 | 1.6 |
| ULBP1 | 3.4 |
| UNC13A | 1.9 |
| VAT1L | 7.1 |
| VCAN | 3.2 |
| VEGFA | 3.5 |
| VEGFC | 1.8 |
| VIP | 1.6 |
| VLDLR | 3.4 |
| WDR66 | 2.4 |
| WNK4 | 1.9 |
| WNT5A | 1.6 |
| XRCC6BP1 | 1.5 |
| ZNF292 | 1.6 |
| ZNF395 | 2.2 |
| ZNF469 | 1.7 |
| ZNF501 | 1.7 |
| ZNF532 | 1.5 |
| ZNF572 | 1.6 |
| ZNF655 | 2.4 |
| ZNF704 | 2.2 |
| ZNF747 | 2.2 |

The relative gene expression was compared with the gene expression of hMVECs precultured in normoxia and stimulated with VEGF-A/TNF-α (n=4).
