# Supplementary material for: Identification of HIF-2α-regulated genes that play a role in human microvascular endothelial sprouting during prolonged hypoxia in vitro
Source: Angiogenesis. 2016 Oct 3;20(1):39–54. doi: 10.1007/s10456-016-9527-4 (PMC5306362; doi:10.1007/s10456-016-9527-4)
Supplement: Supplementary file 3 — Supplementary Table 3. Gene expression data from RNA-sequencing; the HIFα subunits. The relative gene expression in prolonged hypoxia was compared with the gene expression in normoxia, the gene expression with si-HIF-2α and scrambled was compared with hypoxia (untransfected) upon VEGF-A/TNF-α stimulation (n = 4 independent donors). (PDF 202 kb) [file 10456_2016_9527_MOESM3_ESM.pdf]

Supplementary Table 3. Gene expression data from RNA-sequencing; the HIFα subunits.

Gene Name	Hypoxia	Si-HIF-2α	Scrambled
**HIF-1α (HIF1A)**	0.5-fold *	1.3-fold (ns)	1.1-fold (ns)
**HIF-2α (EPAS1)**	0.7-fold *	0.15-fold **	0.75-fold *
**HIF-3α (HIF3A)**	11-fold **	0.25-fold **	0.4-fold **

The relative gene expression in prolonged hypoxia was compared with the gene expression in normoxia, the gene expression with si-HIF-2α and scrambled was compared with hypoxia upon VEGF-A/TNF-α stimulation (n=4).
