# Supplementary material for: Identification of HIF-2α-regulated genes that play a role in human microvascular endothelial sprouting during prolonged hypoxia in vitro
Source: Angiogenesis. 2016 Oct 3;20(1):39–54. doi: 10.1007/s10456-016-9527-4 (PMC5306362; doi:10.1007/s10456-016-9527-4)
Supplement: Supplementary file 4 — Supplementary Table 4. All genes with significantly induced or repressed gene expression upon HIF-2α knock-down in prolonged hypoxic precultured hMVECs after VEGF-A/TNF-α stimulation. The relative gene expression was compared with the gene expression of hMVEC precultured in hypoxia and stimulated with VEGF-A/TNF-α (n = 4 independent donors). (PDF 530 kb) [file 10456_2016_9527_MOESM4_ESM.pdf]

Supplementary Table 4. All genes with significantly induced or repressed gene expression upon HIF-2α knock-down in prolonged hypoxic precultured hMVECs after VEGF-A/TNF-α stimulation.

**Upregulated by si-HIF-2α**		**Downregulated by si-HIF-2α**	
Gene	Fold Change	Gene	Fold Change
AATF	1.5	AASDH	0.60
ABHD5	1.5	ABCF1	0.57
ACSS1	1.6	ABHD14B	0.66
ACTL10	1.6	ACBD6	0.65
ADAP1	2.2	ACER2	0.65
ADCY7	1.6	ACOX1	0.64
ADM2	1.5	ACP6	0.63
ADO	1.5	ACSL3	0.55
AGPAT2	1.6	ACTR5	0.65
AIMP2	1.9	ADH5	0.61
ALKBH4	1.6	AGL	0.57
AOX1,SPATS2L	2.4	AGTPBP1	0.61
AP5B1	1.9	AIG1	0.53
APC2,C19orf25	1.9	AK3	0.50
ARF6	1.5	AKAP9	0.57
ARHGAP8,PRR5,PRR5-ARHGAP8	2.9	AKD1	0.60
ARHGEF2	1.7	AKR7A2,AKR7A3	0.63
ARL16	1.6	ALDH18A1	0.64
ASB7	1.7	ALS2CR8	0.52
ATF5,MIR4751	2.4	AMACR,C1QTNF3,C1QTNF3-AMACR	0.61
ATP1A1OS	1.6	AMN1	0.62
ATXN7	1.6	AMT,NICN1	0.66
ATXN7L2	1.5	AMY2B,RNPC3	0.64
AXIN1	1.7	AMZ2	0.57
BAG1	1.7	ANKH	0.67
BAG3	1.9	ANKRA2	0.67
BAK1	1.8	ANKRD33B	0.62
BCOR	1.8	ANKRD55	0.55
BOP1	1.7	ANO6	0.53
BRAF	1.6	ANP32E	0.57
BRIX1	1.6	ANXA2P2	0.62
BRPF1	1.7	ANXA3	0.67
C11orf61	1.6	ANXA7	0.63
C11orf84	1.5	AP2B1	0.65
C15orf39	1.8	AP3S1	0.62

C16orf5	1.5	APBA1	0.62
C16orf59	1.5	APH1B	0.55
C19orf26	1.8	APLP2	0.65
C19orf66	2.1	ARAP1,STARD10	0.60
C1orf109	1.5	ARG2	0.59
C1orf182	1.8	ARL1	0.64
C1orf51	1.6	ARL2BP	0.57
C1orf52	1.6	ARL3	0.64
C1QBP	1.6	ARL6,EPHA6	0.58
C2orf55	1.7	ARL6IP5	0.59
C3orf15	1.5	ARMC2	0.58
C3orf38	1.7	ARMCX4	0.63
C3orf55	1.5	ARNT	0.62
C4orf43	1.5	ARPC5	0.56
C5orf25	1.7	ARPP19	0.65
C5orf34	1.9	ARRDC3	0.40
C6orf141	2.5	ARSG	0.59
C6orf47	1.5	ASB8	0.62
C7orf26	1.6	ATF2,CHN1	0.66
C7orf43,MIR4658	1.5	ATG10	0.57
C7orf46	1.6	ATG4A	0.59
C8orf33	1.7	ATG7	0.66
C9orf91	1.7	ATP11A	0.64
CACNG8,MIR935	1.6	ATP1B1	0.64
CAMK1D,LOC283070	2.1	ATP5C1	0.66
CAMSAP1	1.7	ATP5E,SLMO2,SLMO2-ATP5E	0.65
CBR3	2.2	ATP8B3	0.64
CCDC59	1.6	ATPBD4	0.66
CCDC9,PRR24,C5AR1	1.8	ATRX	0.51
CDC25B	2.0	B4GALT6	0.66
CDC34	1.7	BAZ2B	0.52
CDCA3	1.7	BCLAF1	0.67
CDKN2A	2.4	BEND5	0.59
CDKN2AIP	1.5	BFSP1	0.60
CDKN3	1.9	BIK	0.56
CDT1	2.4	BIVM,BIVM-ERCC5,ERCC5	0.61
CEACAM19	1.7	BNIP3L	0.52
CEBPB	2.3	BPNT1	0.62
CENPN	1.8	BTBD8,KIAA1107	0.59
CEP192,SEH1L	2.0	C10orf108	0.63
CERS6	1.6	C11orf51	0.64
CGRRF1	1.5	C11orf82	0.58

CIB2	1.7	C14orf101	0.67
CIDECP	1.5	C14orf135	0.67
CLASRP	1.6	C14orf45	0.57
CLCN2	1.5	C15orf62	0.66
CMC2	1.6	C16orf62	0.66
CPEB3	1.6	C1orf63	0.66
CRIP1	2.5	C20orf3	0.31
CRTC2	1.5	C2orf27A,POTEKP	0.55
DEM1	1.6	C2orf74,KIAA1841	0.58
DENND1A	1.9	C3orf64,TMF1	0.61
DENND4B	1.5	C4orf27	0.62
DERL3	1.9	C5orf24,DDX46	0.66
DGCR8,MIR1306,MIR3618	1.6	C5orf54	0.53
DKC1,SNORA56	1.7	C5orf63	0.64
DKFZP586I1420,ZNRF2	1.8	C6orf170	0.66
DLG5	1.5	C6orf70	0.63
DND1	1.5	C6orf72	0.61
DOCK2	1.8	C7orf10	0.49
DPH2	1.6	C9orf125	0.61
DPY19L3	1.5	C9orf80	0.58
DRAP1	1.9	CA12	0.45
DUSP1	2.0	CACNA1C	0.52
EAF2	1.7	CACNB3	0.66
ECHDC3	2.1	CALD1	0.60
EFCAB2	1.5	CAMK4	0.49
EIF2C3	1.5	CASC4,CTDSPL2	0.67
ELF4	2.0	CASD1	0.58
EMP1	1.6	CASP6,PLA2G12A	0.60
EPM2A	1.6	CBR4	0.50
EPS8	1.5	CCBL1	0.60
ERF	1.9	CCDC102B	0.65
ETS2	1.6	CCDC110	0.58
EVC2	1.6	CCDC125	0.62
EXOSC9	2.0	CCDC13	0.64
FAM110A	1.8	CCDC134,SREBF2	0.53
FAM167A	1.7	CCDC23	0.64
FAM46A	1.5	CCDC85A	0.61
FAM86C2P	1.8	CCDC88A	0.65
FANCM	1.6	CCL28	0.57
FARP2	1.7	CCND2	0.60
FBRSL1	1.5	CCNG1	0.65
FBXL14	1.7	CCNG2	0.58
FBXO27	1.6	CCPG1,DYX1C1,DYX1C1-	0.55

		CCPG1	
FBXO6	2.3	CD200	0.54
FJX1	1.9	CD55	0.63
FKBP11	1.6	CD58	0.62
FKBP1B	1.9	CD9	0.56
FKBP5,LOC285847	1.9	CDC123	0.58
FLJ35946	1.8	CDC42	0.54
FLJ41484	2.2	CDH5	0.59
FLNC	1.7	CEMP1	0.66
FOXM1	1.9	CENPBD1	0.60
FOXP4,MIR4641	1.6	CEP19	0.65
FRMD6-AS1	1.7	CEP250	0.61
FRS3	1.7	CEP290	0.64
GATA2	1.8	CEP78	0.58
GATAD2A	1.5	CEPT1	0.66
GBX2	1.9	CETN2	0.52
GDAP1	2.0	CETN3	0.53
GEMIN5	1.6	CHCHD1	0.66
GLIS2	1.8	CHD2,LOC100507217	0.63
GPD2	1.8	CHP	0.61
GPN3	1.6	CHURC1,CHURC1-FNTB,FNTB	0.66
GPR39	1.5	CLCN3	0.59
GPR89B,GPR89C	2.0	CMTM6	0.55
GPRIN1	2.0	CNN3	0.58
GRK6	1.6	CNOT6	0.65
GTF2F1	1.7	CNST,SCCPDH	0.62
GTPBP1	1.6	CNTROB	0.63
HCP5	2.3	COG6	0.59
HES4	3.3	COL4A3BP	0.64
HIRA	1.7	COL5A1	0.66
HIST1H1C	1.9	COLEC10	0.51
HIST1H2AI,HIST1H3H	2.1	COMMD6	0.62
HIST2H2BA	3.5	COX18	0.62
HMGA1	1.9	COX6C	0.62
HMHA1	1.9	CPD	0.53
HOXD4,MIR10B	1.8	CPOX	0.56
HOXD-AS2	2.7	CRBN	0.57
HPSE	2.1	CREBL2	0.59
HSBP1L1	1.6	CRIPAK,KIAA1530	0.66
HSPA14	1.5	CSGALNACT1	0.65
HSPA2	1.9	CTC1	0.66
ICAM4	2.5	CTSO	0.45

IDH3A	1.6	CYP27C1	0.66
IER2	1.9	DAB2	0.63
IER5L	2.1	DAZAP2	0.65
IFITM3	1.6	DCBLD2	0.61
IFNE,MIR31HG	1.8	DDX17	0.61
IFRD1	1.6	DDX6	0.64
IL6	1.9	DEGS1	0.55
IMP4	1.8	DHFRL1	0.63
IRF2BP1	2.0	DHRS4,DHRS4L2	0.65
IRF2BP2	1.6	DIXDC1	0.62
IRF2BPL	1.8	DKFZP564C196	0.67
IRS2	1.8	DLD	0.65
ISL2	2.3	DMD	0.63
ISOC2	1.9	DNAJC25,DNAJC25-GNG10,GNG10,UGCG	0.60
ITPK1	1.9	DNAJC3	0.63
ITPR3	1.5	DNAJC7	0.66
ITPRIP	1.7	DNASE1L1,RPL10	0.56
ITPRIPL1	1.6	DOCK4	0.65
KBTBD8	1.8	DPY30,MEMO1	0.66
KCNS3	2.0	DPYD	0.60
KDM6B,TMEM88	1.6	DSEL	0.61
KHDRBS3	1.5	DSTN	0.60
KIAA0182	2.0	DSTNP2,RPL13P5	0.64
KIAA0226,MIR922	1.9	DSTYK	0.63
KLF11	1.6	DTNA	0.57
KLF2	1.8	DUSP18	0.61
KLHL21	1.7	DUSP19	0.64
KTN1-AS1	1.7	DUSP3	0.66
LAG3	1.7	DYNC1I2	0.61
LIAS	1.5	DYNC1LI2	0.66
LIMA1	1.6	DYNLT3	0.58
LMCD1	2.1	EBLN2	0.57
LMO4	2.0	EEF2K	0.58
LOC100128881	2.1	EFCAB11	0.63
LOC100129138	1.6	EFNB3	0.62
LOC100129387,MIR4712	1.6	EHD2	0.60
LOC100131089	1.7	ELN	0.62
LOC100132247	1.6	EMCN	0.51
LOC100132356	1.7	ENO3	0.63
LOC100132774	1.9	ENSA	0.67
LOC100134713	1.6	ENTPD4	0.61
LOC100286793	2.1	EPAS1	0.15

LOC100288974,MBL1P	1.6	EPB41	0.64
LOC100505894	1.6	EPB41L1	0.57
LOC100506054	1.7	EPM2AIP1	0.60
LOC100506178	1.6	ERLEC1	0.67
LOC100506451	1.5	EXOC6	0.63
LOC100507392	2.1	EXTL3	0.57
LOC100507463	1.7	F2R	0.64
LOC282997	1.9	FADS1	0.44
LOC401010	1.5	FAM102B	0.64
LOC439949	1.6	FAM115C	0.52
LOC613037	1.7	FAM120B	0.65
LOC642846	1.9	FAM134C	0.67
LOC84856	1.8	FAM13A-AS1	0.64
LPIN1	1.6	FAM160A1	0.58
LRCH4,SAP25	1.5	FAM171B	0.58
LRR1	1.5	FAM185A,FBXL13	0.67
LRRC34	1.5	FAM189A2	0.60
LRRC37A3	2.0	FAM20C	0.65
LRRC56	1.9	FAM45A,FAM45B	0.62
LRRK1	1.6	FAM45B	0.67
LYSMD2	1.8	FAM63B	0.67
MAD1L1	1.5	FAM69B	0.66
MAMLD1	1.5	FBXO9	0.66
MAPKAPK5	1.6	FDFT1	0.64
MARS2	1.6	FECH	0.65
MCM5	1.5	FER1L4	0.60
MDK	1.9	FKBP1A,FKBP1A-SDCBP2,SDCBP2	0.67
MERTK	1.7	FKBP7	0.62
METTL1	1.7	FLJ41200	0.57
METTL11A	1.6	FLJ42351	0.65
METTL12	1.5	FLJ46906	0.65
MFSD9	1.6	FLNB	0.58
MICB	1.6	FLOT2	0.66
MIIP	1.6	FNIP1	0.66
MIR1292,NOP56,SNORD86	2.2	FPGT,FPGT-TNNI3K,TNNI3K	0.60
MIR31HG	2.2	FRG1B	0.63
MIR3614,TRIM25	1.6	FRMD6	0.58
MIR4667,TESK1	1.7	FTH1P3	0.51
MLKL	1.6	FTO	0.64
MLL2	1.6	FUT10	0.56
MMP17	1.8	G3BP1	0.56

MOV10	1.6	GALC	0.51
MRM1	1.5	GALNT1	0.62
MSTO2P	1.6	GAS2	0.59
MT1E	1.7	GAS2L3	0.63
MYC	2.0	GBE1	0.64
MYD88	1.6	GCHFR	0.64
MYEOV	2.5	GDF11	0.65
NANOS3	1.9	GEMIN8	0.64
NBR2	1.7	GIN1	0.64
NCDN	1.6	GIPC3	0.60
NCK2	1.6	GK	0.60
NCOA2	1.6	GK3P	0.59
NCOR2	1.6	GLCE	0.54
NDOR1	1.6	GLOD4	0.67
NEK8,TRAF4	2.4	GMCL1	0.61
NEURL3	2.6	GMCL1P1	0.47
NFATC3	1.5	GNA12	0.65
NOC3L	1.6	GNG11	0.61
NOLC1,PPRC1	1.8	GNPAT	0.64
NOP16	1.8	GNPDA1	0.55
NOTCH1	1.5	GNRH1	0.58
NPHP4	1.5	GNS	0.62
NPIP	1.8	GOLGA2	0.67
NPIPL3	1.7	GOLIM4	0.62
NR4A1	2.1	GON4L	0.66
NSUN2	1.7	GPBP1L1	0.60
NSUN5,NSUN5P2	2.2	GPC1	0.61
NSUN6	1.6	GPR160	0.60
NUDT9P1	2.1	GRAMD3	0.55
NUP153	1.6	GSK3B	0.61
NUP160	1.6	H2AFV	0.62
NUP98	1.7	HACE1	0.60
NUPL2	1.5	HADHA	0.61
NXT1	1.5	HADHB	0.61
ORAOV1	1.8	HBP1	0.63
OSGIN1	1.9	HCG11	0.64
PACSIN3	1.7	HERC1	0.66
PAPD4	1.6	HIBADH	0.67
PARP10	1.6	HMG20A	0.57
PARP8	4.1	HMOX2	0.65
PDE4DIP	2.0	HNRNPA2B1	0.66
PDRG1	1.5	HNRNPM	0.63
PDXP	1.6	HSD17B11	0.57

PEMT	2.0	HSDL2	0.60
PHACTR4	1.8	HTATIP2	0.63
PHF15	1.7	HTRA3	0.59
PHF19,TRAF1	2.9	HYAL3,NAT6	0.63
PHLDA2	1.6	IFNAR1	0.60
PLD6	1.6	IFNAR2,IL10RB	0.58
PLEKHF1	2.2	IFNGR1	0.65
PLEKHG3	1.6	IFT27	0.63
PLEKHO2	1.9	IFT80	0.63
PLXNA4	1.7	IFT81	0.54
PNO1	1.8	IK,MIR3655	0.62
POLR3C	1.9	IL13RA1	0.65
POLR3K	1.6	IL1A	0.67
PPCDC	1.6	IL27RA	0.63
PPIF	1.5	IL6ST	0.58
PPM1J	1.9	IMPACT	0.60
PPP3CC	1.7	IMPAD1	0.60
PRICKLE3	1.5	INTS2	0.64
PRKCD	1.6	IPP	0.59
PRKD2	1.6	IPW,SNORD116-17,SNORD116-21,SNORD116-24,SNORD116-26,SNORD116-4	0.60
PRPF4	1.6	IQCC	0.66
PRR3	1.6	IQCK	0.64
PRRX1	1.6	IQGAP1	0.51
PTHLH	2.1	ISCU	0.60
PTK2B	2.1	ISOC1	0.67
PTP4A3	2.7	ITGA11	0.65
PTTG1	1.6	ITGA6	0.59
PUS1	1.8	ITGB1	0.60
PWWP2B	1.7	ITPR2	0.56
RASD1	1.9	JRKL	0.67
RASGRF1	2.0	KATNAL1	0.66
RASL10A	1.7	KBTBD3	0.55
RGMB	1.5	KBTBD6	0.59
RILPL1	1.7	KCNAB1	0.54
RP9,RP9P	1.8	KDELR2	0.60
RPA4	1.7	KIAA0196	0.67
RPL39L	1.7	KIAA0232	0.60
RPL9	1.6	KIAA0317	0.65
RPS6KB2	1.6	KIAA0895	0.62

RPUSD1	1.6	KIAA1211	0.51
RRP1	1.8	KIAA1324	0.58
RRP12	1.8	KIAA1377	0.54
RRP9	2.1	KIAA1407	0.60
RRS1	1.6	KIAA1462	0.66
RTKN	2.1	KIAA1797	0.60
RUSC1	1.5	KIFAP3	0.61
S100A2	1.8	KISS1	0.41
SART1	1.5	KLHL12	0.67
SASH1	1.7	KLHL24	0.50
SBDSP1	1.6	KRT19,KRT33A	0.60
SCAF4	1.5	KRT80	0.60
SCO1	1.5	LAMP2	0.59
SDHAP1	1.6	LCORL	0.64
SDSL	1.9	LDLRAP1	0.65
SERPINB2	1.8	LEPR,LEPROT	0.54
SESN3	1.8	LEPREL1	0.57
SF1	1.8	LEPROTL1	0.53
SFXN4	1.6	LINC00085,MIR125A	0.66
SGTA	1.6	LIX1L	0.56
SIAH2	1.6	LOC100130890	0.56
SIK1	1.6	LOC100131094	0.66
SLC20A2	1.6	LOC100499466	0.61
SLC25A33	1.8	LOC100505495	0.64
SLC43A1	1.6	LOC100505648	0.63
SLC7A5	1.7	LOC100505678	0.63
SNAPC1	1.6	LOC100505761	0.51
SNAPC4	1.5	LOC100506746	0.62
SNHG8	1.7	LOC221442,NFYA,APOBEC2	0.62
SNORD7	1.8	LOC254100	0.64
SOCS3	2.0	LOC286437,MCART6,MIR1256	0.62
SOCS6	1.5	LOC375190	0.66
SOLH	2.0	LOC550643	0.67
SOX18	1.6	LOC642852	0.62
SP140L	1.7	LOC643401	0.48
SPHK1	1.5	LOC646999	0.64
SPOCD1	1.7	LOC728758	0.64
SPRY4	1.5	LOXL2	0.60
SPTBN5	1.6	LPCAT1	0.63
SRD5A1	1.6	LPCAT2	0.60
SRM	2.0	LPXN	0.54

SSBP2	1.6	LRCH3	0.65
STARD8	1.7	LRP12	0.53
STEAP1	1.6	LRRIQ1	0.52
STOML1	1.7	LUST	0.58
SUV39H1	1.5	LYPD3	0.54
TAP2	1.9	MALAT1	0.65
TBC1D30	1.6	MAN1A1	0.66
TBKBP1	1.9	MAP3K1	0.55
TBP	1.5	MAP7D3	0.62
TCF19	1.6	MAPK8IP1	0.65
TCOF1	1.7	MAPKBP1	0.64
TEX2	1.5	MAPRE3	0.66
TFAP4	1.6	MARCH4	0.55
TFEB	2.0	MARCH8	0.62
THAP4	1.6	MB21D2	0.61
TIMM9	1.5	MCU	0.62
TLE4	1.9	MCHR1	0.11
TMCO4	1.8	ME1	0.66
TMEM121	1.7	MED20	0.63
TMEM194B	1.6	MED7	0.58
TMEM201	1.5	MFSD11	0.64
TMEM91	2.3	MGC23284	0.67
TMIE	1.9	MGST1	0.67
TOB1	1.5	MIR331,MIR3685	0.48
TOE1	1.6	MIR4444-1	0.60
TOMM40	1.6	MIR4469,RNF170	0.63
TOP1MT	1.5	MKL2	0.62
TRAP1	1.7	MKS1	0.62
TRIB1	2.0	MLL5	0.64
TRIM11	1.9	MMD	0.47
TRIM62	1.7	MME	0.47
TRIM68	1.6	MMP14	0.67
TUFT1	1.6	MORF4L1	0.55
TXLNG	1.5	MOSPD1	0.60
TYW1B	1.7	MPP4	0.58
UBE2L6	1.6	MPZL1	0.36
UFSP1	1.8	MRC1	0.65
UHRF2	1.5	MREG,PECR	0.59
VTI1A	1.6	MRFAP1L1	0.67
WDFY2	1.6	MRGPRX4	0.51
WDR3	1.6	MRPS28,TPD52	0.67
WDR4	1.6	MRS2	0.61
WDR74	2.0	MSL3P1	0.52

WDR76	1.6	MSMO1	0.62
WDR78	1.5	MTIF3	0.57
WTIP	1.8	MXRA7	0.57
ZBTB42	1.8	MYH10	0.58
ZBTB49	1.5	MYO1B	0.65
ZCCHC18	1.7	MYO1D	0.55
ZCWPW2	1.6	NACA	0.63
ZFP3	1.5	NAP1L1	0.55
ZFP36	2.2	NAP1L5	0.66
ZFP37	2.1	NBAS	0.62
ZNF133	1.8	NBEAL2	0.65
ZNF138	1.9	NBPF16	0.49
ZNF205	1.7	NCK1	0.61
ZNF251	1.6	NDRG3	0.53
ZNF275	1.6	NEDD4L	0.56
ZNF319	1.5	NFATC2	0.65
ZNF324,ZNF324B	1.8	NFIB	0.46
ZNF469	2.0	NHLRC3	0.65
ZNF473	1.7	NIN	0.61
ZNF496	1.6	NIPAL2	0.60
ZNF517	1.5	NLRC3	0.63
ZNF580,ZNF581	1.8	NOVA1	0.41
ZNF582	1.7	NPR2	0.54
ZNF584	1.7	NUAK1	0.65
ZNF597	1.6	NUDT13	0.55
ZNF598	1.9	NUDT9	0.62
ZNF662	1.6	OAT	0.64
ZNF689	1.6	OAZ2	0.66
ZNF696	1.6	OCIAD2	0.52
ZNF746,ZNF767	1.7	ODZ3	0.61
ZNF75A	1.5	OR5B17	0.53
ZNF777	1.9	OSBPL8	0.63
ZNF778	1.5	OSBPL9	0.59
ZNF783	1.6	OSTC,RPL34	0.58
ZNF786	1.7	OSTF1	0.63
ZNF837	1.6	OXSM	0.61
ZRANB3	1.6	P4HA1	0.63
ZSCAN22	1.5	PACS2	0.59
ZSCAN29	1.5	PCCB	0.65
		PCDH10	0.64
		PCGF3	0.64
		PCMTD2	0.59
		PCYOX1	0.47

	PDCL	0.56
	PDE4D	0.66
	PDGFC	0.67
	PDGFD	0.56
	PDHB	0.66
	PDLIM3	0.65
	PEX12	0.65
	PFDN1	0.63
	PGM2L1	0.67
	PHF10	0.56
	PHF6	0.59
	PHKB	0.59
	PHLDB2,PLCXD2	0.66
	PHTF1	0.60
	PIAS1	0.64
	PIGP	0.67
	PIK3CB	0.52
	PIN4	0.67
	PIP5K1C	0.66
	PJA2	0.50
	PKI55	0.64
	PLA2G4A	0.62
	PLDN	0.55
	PLEKHA1	0.62
	PLOD2	0.55
	PLXNB1	0.64
	PMEPA1	0.59
	PMPCB,PSMC2	0.62
	PNPLA8	0.55
	PODXL	0.53
	POPDC3	0.49
	POU2F1	0.63
	PPA2	0.63
	PPARD	0.65
	PPARG	0.49
	PPCS	0.64
	PRICKLE2	0.58
	PRKAR1A	0.66
	PRPF39	0.65
	PRPF4B	0.66
	PRR5L	0.62
	PRRC1	0.60
	PRSS12	0.64

	PSMB1	0.65
	PSMD1	0.66
	PSMD10	0.58
	PSME1	0.67
	PSPH	0.63
	PTCHD4	0.60
	PTK7	0.55
	PTPLAD2	0.54
	PTTG1IP	0.61
	RAB1A	0.64
	RAB30	0.59
	RAB3B	0.56
	RAB3D	0.62
	RAB40A	0.65
	RAB42	0.64
	RAB6A	0.62
	RAB6B	0.61
	RABL2B	0.64
	RAC1	0.61
	RAI14	0.64
	RALA	0.55
	RALGAPB	0.66
	RALGPS2	0.50
	RASSF9	0.37
	RBFOX2	0.64
	RBM4B	0.64
	RCHY1	0.64
	RCN1	0.61
	RDH10	0.58
	REPS2	0.49
	REV3L	0.61
	REXO2	0.61
	RHOA	0.64
	RHOQ	0.56
	RIMS1	0.63
	RMRP	0.57
	RNF130	0.62
	RNF141	0.56
	RNF207	0.58
	RPL11	0.67
	RPL15	0.63
	RPL23	0.66
	RPL27A	0.67

	RPL5	0.64
	RPS13	0.66
	RPS28	0.61
	RPS8,SNORD55	0.65
	RRAGB	0.65
	RYK	0.59
	SARNP	0.65
	SATB1	0.66
	SCD	0.63
	SCD5	0.59
	SDC2	0.57
	SDC3	0.60
	SDCBP	0.62
	SDHC	0.61
	SEC22A	0.64
	SEC62	0.65
	SELT	0.66
	SEPT2	0.60
	SEPT8	0.61
	SERINC1	0.58
	SESN1	0.63
	SESTD1	0.63
	SF3B1	0.59
	SFPQ	0.60
	SKAP2	0.53
	SLC25A40	0.63
	SLC2A11	0.59
	SLC37A3	0.65
	SLC38A4	0.54
	SLC39A10	0.46
	SLC40A1	0.66
	SLC44A1	0.66
	SLC6A15	0.61
	SMAD1	0.66
	SMARCC2	0.65
	SNX13	0.60
	SNX29	0.63
	SNX33	0.60
	SPA17	0.60
	SPAG16	0.57
	SPATA18	0.62
	SPCS1	0.58
	SPG7	0.65

	SPPL3	0.64
	SREK1IP1	0.65
	SSR1	0.66
	ST3GAL6	0.63
	STARD9	0.56
	STC1	0.63
	STK32B	0.65
	SUCLG1	0.66
	SUCLG2	0.66
	SUMO2	0.58
	SWAP70	0.63
	SYNGAP1,ZBTB9	0.66
	SYT9	0.60
	TACC1	0.67
	TAF1L	0.67
	TAF9B	0.59
	TANC1	0.65
	TBC1D15	0.60
	TBC1D5	0.60
	TBC1D7	0.63
	TBCA	0.56
	TBRG1	0.65
	TCEAL4	0.57
	TCF4	0.65
	TCP11L2	0.47
	TEK	0.62
	TGFBR2	0.60
	TGOLN2	0.59
	TIE1	0.64
	TIGD2	0.61
	TIMP4	0.45
	TM2D2	0.66
	TM4SF1	0.65
	TM4SF18	0.53
	TMBIM6	0.59
	TMCO3	0.62
	TMED10	0.56
	TMEM128	0.66
	TMEM14A	0.52
	TMEM14B	0.54
	TMEM14C	0.54
	TMEM154	0.53
	TMEM17	0.61

	TMEM184B	0.62
	TMEM214	0.67
	TMEM50B	0.59
	TMEM67	0.62
	TMEM98	0.66
	TMTC4	0.62
	TNFAIP8	0.57
	TNFRSF10B	0.63
	TONSL,VPS28	0.58
	TOP2B,MIR4442	0.62
	TP53INP2	0.50
	TPCN2	0.66
	TRAK1	0.63
	TRIM16	0.58
	TRIM24	0.63
	TRIM37	0.61
	TRPC1	0.59
	TSC1	0.66
	TSPAN13	0.65
	TSPAN14	0.67
	TSPAN2	0.54
	TSPAN3	0.66
	TSPYL1	0.64
	TTBK2	0.67
	TTC1	0.63
	TTC13	0.57
	TTC3	0.57
	TTC3P1	0.62
	TTC5	0.65
	TUBB3	0.62
	TXLNB	0.67
	TXNDC15	0.56
	TXNDC16	0.59
	UBE2E3	0.60
	UBE2H	0.60
	UBE2W	0.63
	UBQLN2	0.63
	UBXN4	0.56
	UGP2	0.63
	UNKL	0.60
	USP35	0.66
	UTY	0.66
	VCP	0.65

	VDAC1	0.57
	VOPP1	0.64
	VPS41	0.60
	VWA5A	0.61
	WBP5	0.53
	WDR66	0.61
	WNT5A	0.56
	WTH3DI	0.63
	WWC1	0.59
	XRCC6BP1	0.60
	YIPF5	0.65
	YTHDC2	0.52
	ZBTB38	0.63
	ZC3H6	0.63
	ZCRB1	0.56
	ZEB1	0.65
	ZFP14	0.53
	ZFYVE28	0.56
	ZKSCAN3	0.65
	ZKSCAN4	0.62
	ZMAT3	0.53
	ZMYM1	0.66
	ZMYM6	0.61
	ZNF10	0.59
	ZNF175	0.67
	ZNF221	0.56
	ZNF248	0.57
	ZNF266	0.65
	ZNF292	0.59
	ZNF32	0.59
	ZNF33A	0.58
	ZNF33B	0.65
	ZNF345	0.58
	ZNF417,ZNF418,ZNF814	0.60
	ZNF434	0.66
	ZNF548	0.66
	ZNF561	0.65
	ZNF567	0.67
	ZNF571	0.53
	ZNF587	0.56
	ZNF589	0.61
	ZNF624	0.61
	ZNF772	0.62

	ZNF880	0.62
	ZSCAN30	0.67
	ZSWIM3	0.60
	ZSWIM5	0.56

The relative gene expression was compared with the gene expression of hMVEC precultured in hypoxia and stimulated with VEGF-A/TNF-α (n=4).
